# The Cell Cycle–Regulated Genes of *Schizosaccharomyces pombe*

**DOI:** 10.1371/journal.pbio.0030225

**Published:** 2005-06-28

**Authors:** Anna Oliva, Adam Rosebrock, Francisco Ferrezuelo, Saumyadipta Pyne, Haiying Chen, Steve Skiena, Bruce Futcher, Janet Leatherwood

**Affiliations:** **1** Department of Molecular Genetics and Microbiology, Stony Brook University, Stony Brook, New York, United States of America,; **2** Department of Computer Science, Stony Brook University, Stony Brook, New York, United States of America; Lawrence Berkeley LabUnited States of America

## Abstract

Many genes are regulated as an innate part of the eukaryotic cell cycle, and a complex transcriptional network helps enable the cyclic behavior of dividing cells. This transcriptional network has been studied in *Saccharomyces cerevisiae* (budding yeast) and elsewhere. To provide more perspective on these regulatory mechanisms, we have used microarrays to measure gene expression through the cell cycle of *Schizosaccharomyces pombe* (fission yeast). The 750 genes with the most significant oscillations were identified and analyzed. There were two broad waves of cell cycle transcription, one in early/mid G2 phase, and the other near the G2/M transition. The early/mid G2 wave included many genes involved in ribosome biogenesis, possibly explaining the cell cycle oscillation in protein synthesis in *S. pombe*. The G2/M wave included at least three distinctly regulated clusters of genes: one large cluster including mitosis, mitotic exit, and cell separation functions, one small cluster dedicated to DNA replication, and another small cluster dedicated to cytokinesis and division. *S. pombe* cell cycle genes have relatively long, complex promoters containing groups of multiple DNA sequence motifs, often of two, three, or more different kinds. Many of the genes, transcription factors, and regulatory mechanisms are conserved between *S. pombe* and *S. cerevisiae*. Finally, we found preliminary evidence for a nearly genome-wide oscillation in gene expression: 2,000 or more genes undergo slight oscillations in expression as a function of the cell cycle, although whether this is adaptive, or incidental to other events in the cell, such as chromatin condensation, we do not know.

## Abstract 2

A comprehensive examination of gene expression throughout the cell cycle of fission yeast is compared with recent related studies to highlight robust transcriptional patterns.

## Introduction

The yeasts *Schizosaccharomyces pombe* and *Saccharomyces cerevisiae* are excellent organisms for the study of the cell division cycle. Both yeasts have many well-characterized cell division cycle *(cdc)* mutants [1–5], and both have a long history of genetic and molecular cell cycle studies. However, they diverged more than 1 billion years ago, and have many lifestyle differences.

In particular, the two yeasts have different cell cycles. *S. pombe* divides by fission, a symmetrical process in which a septum grows across the center of a long cylindrical cell, dividing the old cell into two equal new cells. Moreover, the main control point in the *S. pombe* cell cycle is a size control in G2, not in G1 as in *S. cerevisiae* and many other organisms. In *S. pombe,* when cells reach a critical size, the Cdc2 protein kinase is activated both by cyclin binding and also by Cdc25 phosphatase removal of the inhibitory phosphate from tyr15 of Cdc2, and this leads to mitosis. Once nuclear division has occurred, the cell moves quickly into S phase without an appreciable G1. Therefore S phase is largely completed by the time cytokinesis/cell separation occurs. Thus, when the cells are growing in good conditions, cells have a long G2, and most cell cycle–specific events are completed in a relatively small portion of the cell cycle encompassing M, G1, and S, with S occurring coincident with cytokinesis. When conditions are poor, a cryptic size control appears in G1 phase; that is, a G1 phase appears and becomes longer as growth rate becomes slower.

In contrast, *S. cerevisiae* divides by “budding,” an inherently asymmetrical process whereby a large mother cell generates a small daughter bud. Once born as a separate cell, the small daughter grows in volume through a long G1, and commits to division at a G1 event called “START.” START involves the activation of a pair of closely related transcription factors, MBF and SBF, and the induction of 100 or more genes. After START, DNA synthesis is initiated, and a bud forms. There is a short G2 phase, followed by mitosis and cytokinesis, and then cells enter the next G1. When cells are growing rapidly in good conditions, G1, S, G2, and M phases are of similar lengths, and so various cell cycle–specific events are distributed somewhat equally around the cycle. However, when cells are growing slowly in poor conditions, almost all the increased length of the cell cycle is accounted for by an increased G1, and most cell cycle–specific events occur over a relatively small percentage of the cell cycle, encompassing “START,” S phase, and mitosis.

Microarrays have been used to analyze gene expression in synchronized *S. cerevisiae*. There are at least 800 genes whose transcripts oscillate as a function of the cell cycle [6]. The cataloging of these transcripts has helped describe what happens in a cell cycle. In addition, because many of the oscillating genes are regulatory, the microarray analysis has helped us understand how the *S. cerevisiae* cycle is regulated. In view of the fact that *S. pombe* also has a well-studied cell cycle and because these two yeasts have both differences and similarities in the way they carry out a cell cycle, it is of interest to characterize oscillating transcripts in*S. pombe* also, to understand at a deeper level what is preserved and what changes across the cell cycles of these two model eukaryotes.

Recently, Rustici et al. [7] and Peng et al. [8] have published microarray analyses of *S. pombe* cell cycle genes. Our results are broadly similar to theirs, but as described below, each group finds a somewhat different set of genes. There is excellent agreement between the groups with respect to the most strongly regulated genes, but naturally there is less agreement for more weakly regulated genes. Here, we concentrate on the 750 genes that are most strongly regulated, but we believe that there may be a total of 2,000 or more genes that have at least weak cell cycle regulation. A large number of weakly to moderately oscillating genes peak in G2 phase, and these are highly enriched for functions in ribosome biogenesis. Our analysis of the cell cycle–regulated promoters shows them to be surprisingly complex, and shows clusters of multiple regulatory motifs similar to clusters of motifs found in the developmental genes of *Drosophila*. Although Rustici et al. [7] have pointed out several differences between the cell cycles of *S. pombe* and *S. cerevisiae,* we find that there are also striking similarities, suggesting deeply conserved mechanisms.

## Results

### Synchronous Cultures and Identification of Cell Cycle–Regulated Transcripts

**Figure 1:**
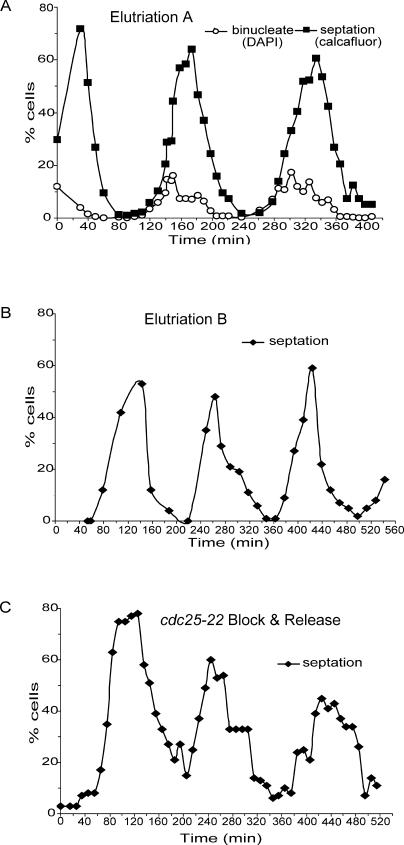
Synchrony (A) Samples from elutriation A were double stained with calcofluor (for septa) and DAPI (for nuclei). Cells were assayed for initiation of anaphase by scoring cells with two nuclei but no septum (binucleates, open circles). The cells were also scored for septation (filled squares). (B) Cells from elutriation B were assayed for septation by phase contrast microscopy. (C) Cells from the *cdc25–22* block release were assayed for septation by phase contrast microscopy.

Three synchronous cultures were studied, one generated by *cdc25* block release, and two generated by elutriation. Each culture was sampled through three cell cycles, giving nine cell cycles of data. Synchrony and cell cycle position were assayed by scoring initiation of anaphase and septation microscopically (Figure 1). RNA was extracted, converted to cDNA, labeled, and hybridized to arrays, and then fluorescence was analyzed. Gene expression was assayed as a ratio of experimental cDNA to asynchronous control cDNA. In total, approximately 1.2 million data points were generated from cell synchrony experiments. Fourier analysis identified cyclic expression patterns. Monte Carlo simulations were used on shuffled expression-ratio data, and compared to the actual, cyclic data, to generate a *p-*value for the cyclicity of each gene. These *p-*values ranged from less than 10^−16^ for the most cyclic genes (i.e., the probability that the observed oscillation occurs by chance is less than 10^−16^), to 0.997 for the least cyclic gene of the 5,000 studied. We ranked all 5,000 genes by *p-*value, with the most significantly oscillating genes at the top. The amplitude of the oscillation is a major contributor to the *p-*value, so genes with higher amplitude oscillations tend to rank higher than genes with lower amplitudes.

The list of all 5,000 genes ranked by *p-*value and other associated information such as time of peak expression is given in Table S1. The raw data have been deposited at ArrayExpress (http://www.ebi.ac.uk/arrayexpress/). The raw data, all figures and all tables, are available at: http://publications.redgreengene.com/oliva_plos_2005/.

**Figure 2:**
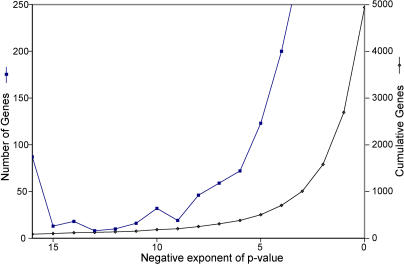
Distribution of *p-*Values for Cell Cycle–Regulated Genes The *x*-axis shows bins of *p-*values of the significance of cell cycle regulation. From the left, the bins are as follows: (1) Genes with *p-*values less than 10^−16^ (87 genes); (2) Genes with *p-*values between 10^−15^ and 10^−16^ (13 genes); (3) Genes with *p-*values between 10^−14^ and 10^−15^ (13 genes); (4) Genes with *p-*values between 10^−13^ and 10^−14^ (eight genes); etc. The number of genes in each bin is shown on the left *y*-axis (dark blue squares). Also shown (right *y*-axis, magenta diamonds) is the cumulative number of genes at each *p-*value or lower. Thus there are about 1,000 genes with a *p-*value of 10^−3^ or less.

The distribution of genes versus *p-*values is shown in Figure 2. There is no clear distinction between “cyclic” and “non-cyclic” genes. Rather, after the best 203 genes, there are simply more and more genes as one goes to poorer and poorer *p-*values.

Because the distribution of genes versus *p-*values continuously increases after gene 203, one must choose a somewhat arbitrary threshold for discussion of cell cycle–regulated genes. We have chosen to discuss the best 750 genes in our *p-*value list. This number is similar to the number of genes chosen by Peng et al. [8] and Rustici et al. [7] as being cell cycle regulated (747 and 407, respectively), and similar to the number of genes chosen for the yeast *S. cerevisiae* (800) [6], thus facilitating comparison of these gene sets. In the vicinity of the 750th gene (and even below), most genes display an oscillatory behavior to the eye, at least in one or two of the three experiments. Finally, the number 750 is obviously somewhat arbitrary, and indeed we have no basis for anything other than an arbitrary cutoff. Because the list of genes is ranked, other investigators may choose their own sets of oscillatory genes from our*p-*value list (Table S1) by choosing any desired cutoff. For the top 750 genes, the false discovery rate is 0.00022, so on a statistical basis, less than one false positive is expected in the list of 750.

Although we will discuss primarily these 750 best genes, there are many more genes that appear to oscillate slightly. A total of 2,262 genes (nearly half the genes in the genome!) have a *p-*value less than 0.05, the usual statistical cutoff. Based on the false discovery rate, we would expect about 53 of these to be false positives, but even so, this leaves well over 2,000 genes with a slight but statistical oscillation.

Previously, 37 cell cycle–regulated genes have been reported in*S. pombe;* 29 of these (78%) are in our top 750. Of the eight that are not in our top 750, two are in the top 1,000. The remaining six *(cdc19/mcm2, cmk1, dmf1/mid1, ppb1, uvi22/rrg1,* and *suc22)* are also not in list of 407 of Rustici et al. [7], and three of these *(cdc19/mcm2, cmk1,* and*ppb1)* are also not in the list of Peng et al. [8]. Thus, these genes are probably quite weakly regulated (except for *suc22,* for which there are two transcripts, one regulated and one not [9]).

**Figure 3:**
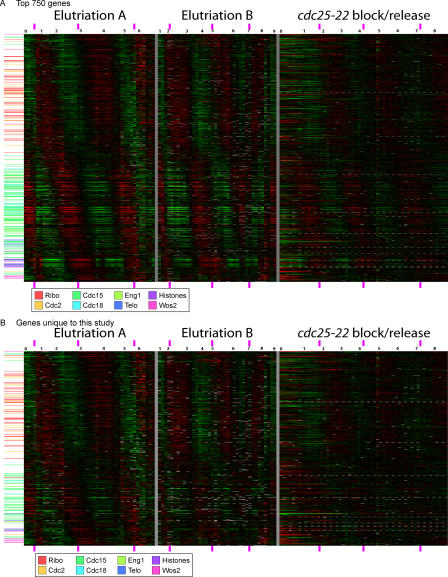
Cell Cycle–Regulated Genes Ordered by Time of Peak Expression (A) Expression data for the top 750 genes is shown, with genes ordered by time of peak expression. Every row represents a gene; every column represents an array from a time-course experiment. Red signifies up-regulation (i.e., an experiment/control ratio greater than one); green signifies down-regulation (i.e., an experiment/control ratio less than one). Black is a ratio close to one, and grey is missing data. Dynamic range is 16-fold from reddest red to greenest green. The time in hours since the beginning of the time course is shown in black numerals at the top of Figure 3. The peaks in septation index are marked with purple rectangles at the top and bottom of the figure. Genes from defined clusters are marked on the left by colored lines, according to the cluster color code shown at the bottom of the figure. (B) As (A), but only the 514 genes found in our study but not found by Rustici et al. [7] are shown.

The top 750 genes are shown in Figure 3 in order of time of expression (i.e., ordered by cell cycle phase). About halfway down this phasogram is an apparent discontinuity; this corresponds to the mid to late G2 trough, when there are relatively few cell cycle–regulated genes (see below).

**Figure 4:**
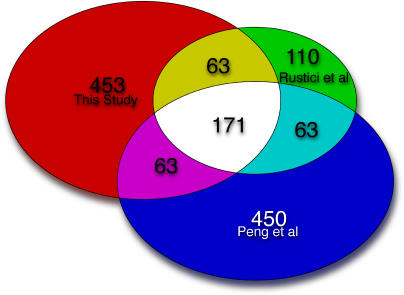
Overlap between Cell Cycle Microarray Studies, by Number of Genes A Venn diagram of the overlap between the three lists of cell cycle–regulated genes from this study, Rustici et al. [7], and Peng et al. [8]. The number of genes in each of the three lists is 750, 407, and 747, respectively. A few genes are not accounted for because of ambiguities in nomenclature.

Rustici et al. [7] have recently compiled a list of 407 periodically-expressed *S. pombe* genes, and while our manuscript was in review, Peng et al. [8] identified 747 similar genes. A comparison of the three studies is shown in Figure 4. The total number of genes found to oscillate in at least one study is 1,373. Of these, 1,013 were unique to just one of the studies, whereas 360 were found in two or three studies, and 171 were found in all three.

Despite the fact that 1,013 genes were found in only one of the three studies, we believe that most of these 1,013 do indeed oscillate to some extent. There are two lines of evidence. First, most of the genes do display a clear oscillatory pattern to the eye, at least in one of the studies. For instance, Figure 3B shows the 516 genes found by us but not by Rustici et al. (63 of these were also found by Peng et al., but the remainder were unique to us). At least in part, we found these genes because our elutriated cells were more synchronous than those of Rustici et al. (compare our Figure 1 to Figure 1B in the supplemental data of Rustici et al.[7]), thus allowing detection of genes with moderate amplitudes.

**Figure 5:**
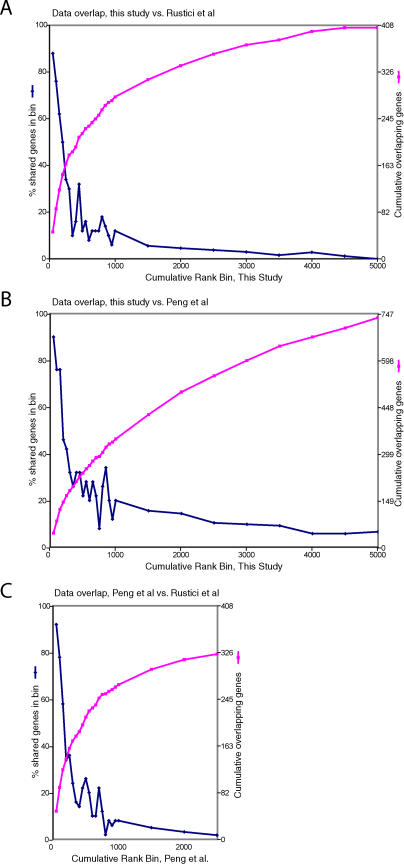
Overlap between Different Cell Cycle Microarray Studies, by Rank (A) Our ranked list of cell cycle–regulated genes is divided into consecutive sets, or bins, of 50 genes. For each set of 50 genes, the number of genes in that set also found in the list of 407 cell cycle genes of Rustici et al. [7] is plotted on the left *y*-axis. For instance, of our best 50 genes, 44 (88%) are found in the list of of 407 genes of Rustici et al., and of our next-best 50 genes, 38 (76%) are also in their list. For the top 15 bins (750 genes), every bin of 50 genes is represented. Afterward, the number plotted represents an average over several bins. The cumulative number of genes in the list of 407 is plotted on the right *y*-axis. (B) As (A), but the bins in our study are compared to the list of 747 genes of Peng et al. [8]. (C) As (A), but the ranked list of Peng et al. is divided into bins, and compared to the list of 407 of Rustici et al. Because Peng et al. ranked only their top 2,700 genes, the graph is truncated after gene 2,700, and the cumulative number of genes rises to only 325.

The second line of evidence is that most of the 1,013 genes unique to one study also display some statistical oscillatory behavior in one or both of the other studies, even though this behavior is not strong enough to surpass the threshold for inclusion on the cell cycle list in those studies. This effect is shown in Figure 5. As might be expected, the top genes in our ranked list and the ranked list of Peng et al. [8] show excellent (approximately 85%) agreement with Rustici et al. [7]. The degree of agreement then drops as one proceeds down the ranked lists. But genes below rank 750 but above rank 2,500 in either list are much more likely to be in the list of Rustici et al. than are genes below rank 2,500. In other words, a gene unique to the study of Rustici et al. is likely to show some oscillatory behavior in the other two studies (i.e., be in the top half of the lists). Analogous comments apply to the genes unique to us, and genes unique to Peng et al. [8].

Before the publication of Peng et al. [8], we had compared our study to that of Rustici et al. [7] to look for discrepancies. We identified a total of 21 genes (11 from Rustici et al., ten from us) that appeared very strongly regulated in one study, but not at all regulated in the other. We have now checked these 21 genes against the results of Peng et al., and find that 17 of the 21 appear regulated in Peng et al., whereas four (three from us and one from Rustici et al.) do not appear regulated. Thus it seems that both we and Rustici et al. have been conservative in our identification of cell cycle–regulated genes and tend to get false negatives rather than false positives.

In summary, the three cell cycle lists together implicate about 1,300 genes, and our ranked *p-*value list does not become worse than a *p-*value of 0.05 until gene number 2,262. We believe that a very large number of *S. pombe* genes, 2,000 or more, have at least a weak cell cycle oscillation.

### Two Genome-Wide Waves of Transcription

**Figure 6:**
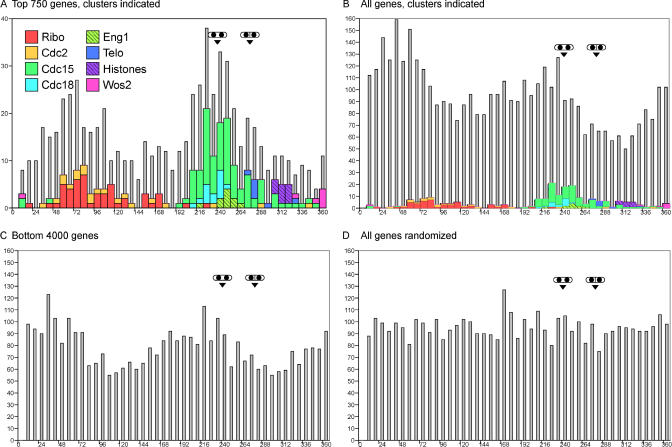
The Number of Genes Peaking during Each Portion of the Cell Cycle The cell cycle was divided into 45 consecutive portions, or bins. If the cell cycle is considered as a circle of 360°, then each bin occupies 8°. Every gene was analyzed using a Fourier transform to determine the time of peak expression in elutriation A, from 0° to 360°. The number of genes peaking in each bin was summed and plotted (grey bars, background), with the number of genes in each bin shown on the *y*-axis. Genes in specific clusters are shown by colored bars (foreground). The Fourier transform calculation was similarly used to derive the time of peak septation and peak binucleate cells (from Figure 1) and these cell cycle landmarks are indicated. (A) The top 750 cell cycle–regulated genes were analyzed for time of peak expression. Genes from different clusters are “stacked” when they occur in the same bin. (B) All genes (∼5,000) were analyzed for time of peak expression, exactly as in (A). (C) The bottom 4,000 genes were analyzed for time of peak expression. Data was extracted from arrays before the red/green normalization step, so that the bottom 4,000 genes would not be affected by the normalization and the cyclic expression of the top 750 genes. (D) All genes (∼5,000) were analyzed for time of peak expression after genewise random shuffling of microarray observations. This randomization serves as a negative control for the Fourier calculation in parts (A), (B), and (C).

To examine the distribution of gene expression around the cycle, Fourier analysis was used to determine the time at which each gene's expression peaked (the “phase angle” of peak expression). For genes in the bottom half of the 5,000 gene rank list (i.e., genes that did not cycle appreciably), phase angles were largely determined by noise, but nevertheless would tend toward the peak of any weak cyclic behavior that may have existed. The number of genes peaking at each time in the cycle was plotted (Figure 6) for four groups of genes: the most-regulated 750 genes (Figure 6A), all genes (Figure 6B), the least regulated 4,000 genes (Figure 6C), and all genes after random shuffling of ratio data (Figure 6D). The peaks of septation and binucleates were also determined by Fourier analysis. (See Materials and Methods for information on red/green normalization.)

There were two striking findings. First, it appears that there are two broad waves of gene expression, one peaking in early to mid G2, and the second peaking in late G2/M, whereas there are troughs in mid to late G2, and in S. The early/mid G2 peak contains the Ribosome biogenesis cluster (see below) and associated genes, whereas the late G2/M peak contains the genes of the Cdc15, Cdc18, and Eng1 clusters (see below), which are important for M and S. Second, the two waves of gene expression were seen even in the 4,000 least-cyclic genes. As noted above, there is statistical evidence from *p-*values that 2,000 or more genes may oscillate slightly. The two waves of expression seen for the bottom 4,000 genes confirm that many of these genes do indeed oscillate. If the fluctuations in these 4,000 genes had simply been due to noise, then the peak phase angles would have been uniformly distributed from 0° to 360° (as confirmed by repeating the analysis on shuffled data; Figure 6D). Combined with the evidence of the *p-*values, this analysis suggests that many, or most (or possibly all!) of the least-cyclic 4,000 genes do in fact oscillate slightly, and that there are two nearly genome-wide peaks in gene expression. These peaks might represent periods when the cell is preparing for a high level of cell cycle–specific activity, or when transcription (of any kind) is activated on a genome-wide basis. Alternatively, one might focus on the troughs, which might represent periods with little cell cycle–specific activity, or periods when transcription is repressed on a genome-wide basis (see Discussion).

### Cluster Analysis

To study the regulation of the cell cycle, we wished to find clusters of co-regulated genes potentially responding to the same transcription factor. However for this purpose it is not sufficient to find genes expressed at the same time, because such genes might be responding to different mechanisms of regulation. This is an acute problem in *S. pombe,* because mitosis, DNA synthesis, and cytokinesis all occur in a small window of the cell cycle under standard growth conditions.

Therefore our analysis included not only our three time courses of synchronous cells, but also eleven other array experiments that more directly addressed regulatory mechanisms. These experiments (see Materials and Methods) included small cells grown in poor nitrogen to induce a G1 phase; a *cdc10-M17* block-release experiment, to separate S phase events from cytokinesis and septation events; an arrest at G1 (using *cdc10-M17,* encoding MBF transcription factor subunit); an arrest at S (using *cdc22-M45,* encoding ribonucleotide reductase); an arrest at late G2 (using *cdc25–22,* encoding the phosphatase that activates Cdc2); an arrest at M (using *nuc2–663,* encoding a subunit of the anaphase promoting complex); and finally, from the data of Rustici et al. [7], experiments using a constitutively active allele of *cdc10* *(cdc10-c4)*, null and over-expressor alleles of the forkhead transcription factor *sep1,* and null and overexpresser alleles of the transcription factor *ace2*.

Hierarchical clustering was used [10] because the underlying structure of a gene regulatory network is somewhat hierarchical. Thus, a hierarchy found by the clustering algorithm is often interpretable in terms of a hierarchical transcriptional network existing in the cell (see S. J. Gould's essay [11], “Linnaeus's Luck?”, for an illuminating discussion of this issue in a different context http://www.findarticles.com/p/articles/mi_m1134/is_7_109/ai_65132190).

**Figure 7:**
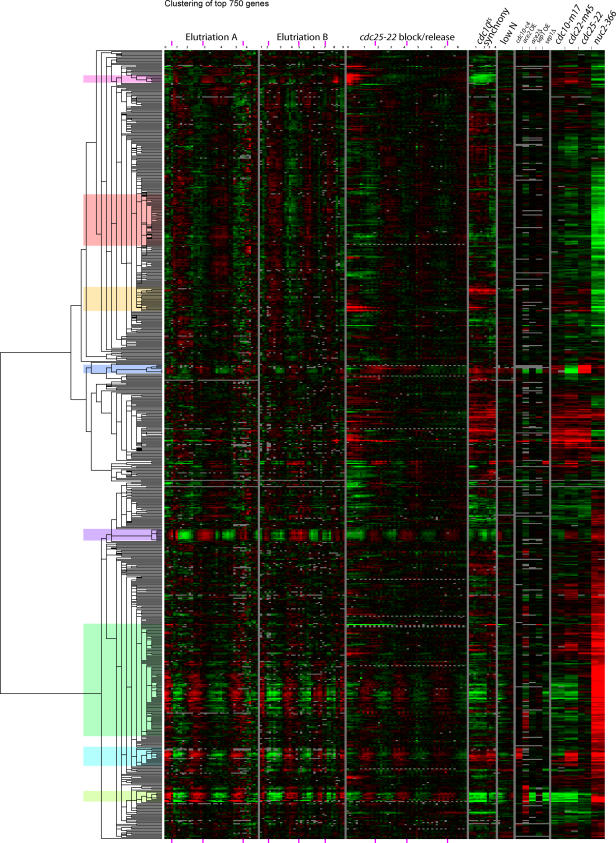
Cluster Analysis of the Top 750 Cell Cycle–Regulated Genes Gene expression data from all experiments were clustered by a hierarchical method (Eisen et al. [10]). Every row represents a gene; every column represents an array. Red signifies up-regulation (i.e., an experiment/control ratio greater than one); green signifies down-regulation (i.e., an experiment/control ratio less than one). Black is a ratio close to one, and grey is missing data. Dynamic range is 16-fold from reddest red to greenest green. The time in hours since the beginning of time-course experiments is shown in black numerals at the top of the Figure. Peaks in septation index are marked with purple rectangles at the top and bottom of the Figure. Clusters discussed in the text are marked with blocks of color. Data for the *cdc10-C4* (asynchronous cells with the hyperactive allele *cdc10-C4*), *ace2* OE (asynchronous cells over-expressing *ace2*), *ace2Δ* (asynchronous *ace2Δ* cells), *sep1* OE (asynchronous cells over-expressing *sep1*), and *sep1Δ* (asynchronous *sep1Δ* cells) are taken from Rustici et al. [7]. *cdc10* encodes a component of the MBF transcription factor; *ace2* encodes the Ace2 transcription factor, and *sep1* encodes a forkhead transcription factor. Other experiments are described in Materials and Methods.

The clustergram of 750 genes is shown in Figure 7. (Treeview files are available as Dataset File S1). We chose eight clusters for analysis and discussion, on the basis that the genes in these clusters are particularly tightly co-regulated. Most of the clusters are named for one representative gene. The clusters are, from the late G2/M wave, the Cdc15, Cdc18, and Eng1 clusters; from S and early G2, the telomeric, histone, and Wos2 clusters; and from the early to mid G2 wave, the Ribosome biogenesis and Cdc2 clusters.

If the genes in each cluster are truly co-regulated, then the promoters of these genes will be bound by the same transcription factor, and therefore the promoters should share a common DNA sequence motif corresponding to the transcription factor binding site. We searched for such motifs upstream of the genes in each cluster. We used three motif search programs: AlignAce, a Gibbs-sampling algorithm [12]; SPEXS, a word-count algorithm (http://www.egeen.ee/u/vilo/SPEXS/) [13,14], and MEME, an expectation-maximization algorithm (http://meme.sdsc.edu/meme/website/intro.html) [15,16]. In general, all three programs found the same motifs.

In the study of Rustici et al. [7], four clusters were found. It is difficult to compare the clusters of Rustici et al. with ours: The genes, experiments and clustering methods were different. However, in general, the clustering of Rustici et al. tended to produce fewer, larger clusters, and focused on time of expression as the main distinction between the clusters, whereas our method produced more, smaller clusters, and focused on regulatory mechanisms (as well as time of expression). Peng et al. [8], like us, used hierarchical clustering and found eight clusters, some of which are quite comparable to ours. However, again, we put more emphasis on regulatory mechanisms as opposed to time of expression, and this generated some different clusters.

### The M Clusters

**Figure 8:**
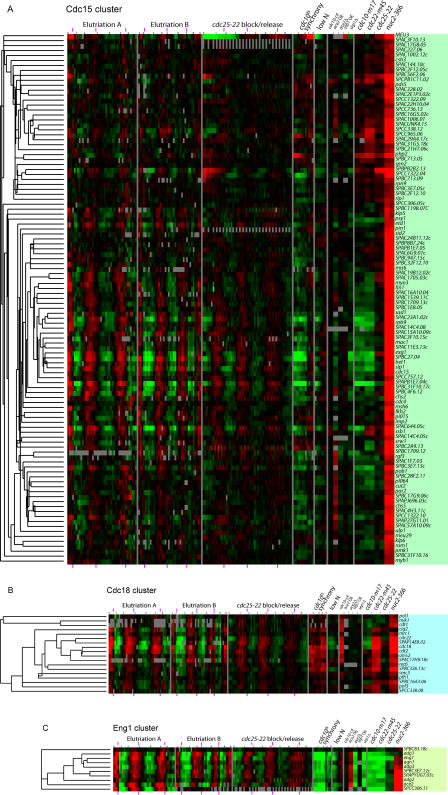
M Phase Clusters Clusters of apparently co-regulated genes were chosen from Figure 7. See legend to Figure 7 for further information. (A) Cdc15 cluster, (B) Cdc18 cluster, and (C) Eng1 cluster.

The wave of expression in the late G2 and M phases includes most of the strongly regulated genes. This wave contains three major clusters, which we call the Cdc15, Cdc18, and Eng1 clusters (Figure 8). Functionally, these clusters are important for mitosis and cell separation, DNA synthesis, and cytokinesis, respectively. Genes of the Cdc15 and Cdc18 clusters peak almost simultaneously with anaphase (see Figures 6 and S1), whereas the Eng1 genes peak slightly later.

The Cdc15 cluster (Figure 8A) is the largest of the three clusters and contains over 100 genes. These are involved in mitosis and mitotic exit, cytokinesis and septation, vesicle trafficking, cell wall remodeling, and other functions. Genes involved in mitosis and mitotic exit include the APC adaptor subunits *srw1* and *slt1,* the prolyl isomerase *pin1, spo12,* the Cdk inhibitor *rum1,* five genes related to ubiquitination, four microtubule-related genes including kinesins *klp5* and *klp6,* and four genes for chromosome segregation.

Cytokinesis/septation fuctions can be ascribed to at least 13 genes including the key SH3 domain gene *cdc15* and its paralog *imp2,* and a third SH3 domain gene, *pob1*. Also present are the kinases *fin1* and *sid2* and phosphatase subunit *par2,* which regulate the septation initiation network. *mob1,* which interacts with *sid2,* is also cell cycle regulated with similar timing, but lies outside the cluster as defined here. Other members likely involved in cytokinesis include genes for the rho family member *rho4,* the putative rhoGEF *rgf3,* the septin *spn2,* and the myosin *myo3*.

Construction of the septum involves synthesis of plasma membrane and deposition of proteins into that membrane. The Cdc15 cluster is rich in proteins involved in these processes. The cluster includes *gwt1,* likely involved in GPI anchor synthesis, and SPAP27G11.01, SPCC306.05c, and SPBC2F12.05c, linked with sterol functions. SPAC227.06 (a predicted Rab interactor), *psy1* and *bet1* (SNAREs), and SPBC31F10.16 are likely to function in vesicle transport. The budding yeast homolog of SPBC31F10.16, *CHS6,* is important for movement of chitin synthase from the trans-Golgi network/endosome to the plasma membrane. Other genes encode cell surface glycoproteins, such as the gene *mac1,* which is localized at poles and septum and is important for cell separation.

Genes for cell wall metabolism include two chitin synthase homologs, a putative chitin synthase regulator, six putative sugar/starch hydrolases, and the MAP kinase *pmk1*.

Finally, diverse other functions are represented. There are at least five genes involved in transcription, most notably the transcription factor *fkh2,* which may be one of the regulators of the Cdc15 cluster [17] (see below). There are also multiple genes involved in mitochondrial functions and in glycosylation.

The three motif search programs all found the consensus motif GTAAACAAA, easily recognizable as a binding site for a forkhead (FKH) transcription factor. Almost every gene in the cluster had such a motif. In *S. cerevisiae,* the main clusters of mitotic genes are also regulated (in part) by forkhead transcription factors. *S. pombe* has several forkhead transcription factors, but the two most likely to regulate the Cdc15 cluster are *sep1* and/or *fkh2*. *sep1* does not oscillate noticeably in our dataset, but it does have phenotypes that could be due to defects in the expression of genes of the Cdc15 cluster, and Rustici et al. [7] have shown defects in cell cycle expression in *sep1* mutants. *fkh2* does oscillate, and is a member of the Cdc15 cluster. The *fkh2* promoter contains two sites each for Forkhead, Ace2, and Cdc10. Interestingly, peak expression of *fkh2* precedes the peak of 94% of the other genes in the cluster, consistent with the idea that it might help regulate these other genes. No direct binding of either Sep1 or Fkh2 to any of these promoters has been demonstrated, and we believe it is still an open question which protein regulates this cluster. It is possible that both proteins contribute. Because forkhead transcription factors can both repress and activate, and because they are regulated both transcriptionally and post-transcriptionally, the regulatory mechanisms could be complex.

The motif search programs also found CCAGCC (Ace2 binding sites) and ACGCG (MBF/Cdc10 binding sites) in a substantial minority of the genes of the Cdc15 cluster. Many genes (e.g., *fkh2* and *pds5*) had all three kinds of sites. MEME (but not the other programs) also found the motif (A/T) TGACAAC. This is probably the same as the motif CATG(A/T) CAAC found by Rustici et al. [7] and named “New 1.” To minimize confusion, we will refer to our version of the motif as “New1v” (“v” for variant).

MEME also found the motif CC(T/A)CG(T/C)TCC, and this may be a variant of the motif (A/T)ACC(T/A)CGC(T/A) (“New 3”) found by Rustici et al. We will refer to our motif as “New 3v.” New 3v was found preferentially in front of genes for cell wall metabolism, such as hydrolases, glycoproteins, chitin synthases, and their regulators. Other functionally related genes are found in the Eng1 cluster (see below), where they appear to be regulated by Ace2. Interestingly, the consensus site for Ace2 ( CCAGCC) is reminiscent of the core of New 3v ( CCACGC), suggesting that an unknown Ace2-like factor could be involved.

We did not find the “PCB” consensus ( GCAAC(G/A)), previously implicated in the control of some of the genes of this cluster [18,19].

The Cdc18 cluster (Figure 8B) contains 18 genes involved in DNA replication. Included in the cluster are*cdc18* (initiation of DNA synthesis),*pol1* (DNA polymerase alpha), *cdt1* (initiation of DNA synthesis),*cig2* (S phase cyclin), *mrc1* (S phase checkpoint), *cdc22* (ribonucleotide reductase), *cdt2* (DNA replication), *smc3* (cohesin), and *pif1* (DNA helicase). These genes are strongly regulated. Peak expression occurs at about the same time as that of the Cdc15 cluster, and is essentially simultaneous with the peak in binucleates (e.g., Figure S1).

The Cdc18 cluster has a very similar cluster in *S. cerevisiae,* called the CLN2 cluster. Both clusters contain genes involved in DNA replication, and both clusters appear to be regulated by the MBF transcription factor (see below). For the Cdc18 *(pombe)* and CLN2 *(cerevisiae)* clusters, many of the genes in the clusters are orthologs; e.g., *mik1/SWE1, cig2/CLB5, mrc1/MRC1, cdc22/RNR1,* and*smc3/SMC3*. Thus the cell cycle clusters regulating DNA synthesis are very highly conserved, with the overall function of the clusters, the regulation of the clusters, and the genes in the clusters, all being quite similar from *S. cerevisiae* to *S. pombe*.

The three motif search programs found two motifs in the Cdc18 cluster: ACGCG, and ACGCG(A/T) CGCG. The first of these is easily recognizable as the binding site for the MBF transcription factor (also known as DSC1) [20–22], whereas the second is a related motif that may be a tandem, double binding site for MBF, or for an MBF-like factor. Consistent with the idea that MBF is a major regulator of this cluster, the genes of the cluster are up-regulated by the *cdc10-c4* mutation (see Figure 8B, and see Rustici et al. [7]) which creates a constitutively active form of MBF. Furthermore, six of these genes are known to be regulated by MBF (*cig2, cdt1, cdt2, cdc18, cdc22,* and *mik1;* GeneDB, Sanger Centre).

*S. cerevisiae* has two MBF-like transcription factors. One is itself called MBF and consists of the DNA-binding protein Mbp1 complexed with the modulatory protein Swi6. The second factor is called SBF and consists of a second DNA-binding protein, Swi4, complexed with Swi6. *S. cerevisiae* MBF and SBF, with their related but distinct DNA-binding proteins, bind to related but distinct motifs, and control the cell cycle expression of partially overlapping sets of genes [23,24]. In *S. pombe,* there is likewise one modulatory protein, Cdc10 (the ortholog of Swi6) and two DNA-binding proteins, Res1 and Res2 (possible orthologs of Mbp1 and Swi4) [20–22,25–27]. Some investigators believe that in *S. pombe,* there is a unique MBF transcription factor and that it contains Cdc10, Res1, and Res2 [25,26,28]. However, other investigators believe that the situation is similar to that found in *S. cerevisiae* and that there may be two MBF-like factors, one containing Cdc10 and Res1, and the other containing Cdc10 and Res2 [27,29]. Although our results do not speak directly to these models, the fact that we find two kinds of motifs is easier to interpret in terms of a model with two different but related forms of MBF.

The Eng1 cluster (Figure 8C) contains nine genes, and these are involved in cell separation. The genes are *adg1* and *adg2* (cell surface glycoproteins), *adg3* (β-glucosidase), *agn1* and *eng1* (glycosyl hydrolases), *cfh4* (chitin synthase regulatory factor), *mid2* (an anillin needed for cell division and septin organization), *ace2* (a cell cycle transcription factor), and *SPCC306.11,* a sequence orphan of unknown function. The genes are very strongly cell cycle regulated. Peak expression of most of the genes occurs slightly later than the genes of the Cdc15 and Cdc18 clusters. Motif searches showed that each gene of the cluster has at least one binding site for the Ace2 transcription factor (consensus CCAGCC). In fact, eight of the nine gen-+-es contain multiple Ace2 binding sites. The exception is the *ace2* gene itself, which contains only one Ace2 binding site, but multiple FKH binding sites. Interestingly, the *ace2* gene is expressed earlier than the other genes of the cluster, consistent with the idea that it might regulate the other genes. The genes are up-regulated when*ace2* is over-expressed, and down-regulated when *ace2* is deleted (see Figure 8C; Rustici et al. [7]). Ace2 was previously shown to be a regulator of *eng1* [30] and *agn1* [31].

The Eng1 cluster has a recognizably similar functional cluster in *S. cerevisiae,* the SIC1 cluster [6]. This cluster also has many genes involved in cell separation (e.g., *EGT2,* an endoglucanase; *CTS1,* an endochitinase; *YGL028c,* a glucanase; *DSE2,* a glucanase; and *CHS1,* a chitin synthase), and the genes of the *S. cerevisiae* cluster are also regulated from Ace2 binding sites of the same consensus sequence ( CCAGC). However, there is only one gene that is clearly present in the cluster in both species, the glycosyl hydrolase *eng1* in *S. pombe,* and its ortholog *DSE4* in *S. cerevisiae*. Thus the overall function of the cluster (cell separation), the nature of many of the enzymes in the cluster (carbohydrate hydrolytic), and the mechanism of gene regulation (binding by Ace2) have been conserved, even though the individual genes in the cluster have been largely shuffled. It is easy to understand why the individual genes are different, because the two species have cell walls containing different carbohydrates (and so requiring different hydrolytic enzymes), and because the modes of cell separation are very different (fission vs. budding). In fact, given these differences, it is remarkable that the mode of regulation and the functional cluster seem to have been conserved.

### The S/Early G2 Clusters

**Figure 9:**
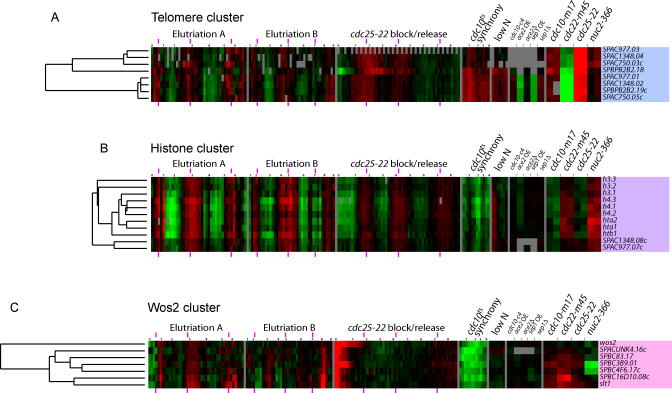
S/Early G2 Clusters (A) Telomeric cluster, (B) Histone cluster, and (C) Wos2 cluster.

The relatively few genes that peak in late M, S, or early G2 fall into three small clusters: the telomeric cluster, the histone cluster, and the Wos2 cluster (Figure 9).

The telomeric cluster (Figure 9A) contains eight tightly clustered genes found near telomeres. Peak expression is in early S. Two of the genes are at telomere 1L; two at 1R; two at 2L, and two at 2R. Interestingly, *S. cerevisiae* also has a cluster containing only telomeric genes (the Y′ cluster), and the genes of that cluster also peak in late G1 or early S.

The histone cluster (Figure 9B) contains all nine histones of *S. pombe*. These are tightly co-regulated and strongly periodic, and form a very tight cluster. Presumably, peak expression of the histone genes marks the time of S phase. These genes are expressed about 30 min after the DNA synthesis genes of the Cdc18 cluster. Surprisingly, the histone cluster contains two non-histone genes, *SPAC977.07c* and *SPAC1384.08c*. These two genes are near telomeres and are homologs of each other, but have no known function. Possibly they are actually co-regulated with the genes of the telomeric cluster, which peak just before the histone cluster.

Motif searches showed that all the histone genes (but not the two telomeric genes) had the motif GGGTTAGGGTT(T/G). A degenerate second copy was sometimes also present. This motif has been noted previously [32]. In addition, six of the histone genes (and both telomeric genes) had a motif similar to an MBF binding site, G(C/G)(T/G) ACGCG.

In *S. cerevisiae,* the histone genes have at least three semi-redundant regulatory systems: First, they have the HIR gene system that represses histone expression outside of S [33,34]. Second, they have regulated mRNA stability, such that the messages are only stable during S [35]. Third, they have a system for gene induction during S. Recently, it has been suggested that this positive system relies on the SBF transcription factor, possibly in combination with a forkhead transcription factor [36]. The fact that an MBF motif is found in front of most of the *S. pombe* histone genes is consistent with the SBF motif found in front of most of the *S. cerevisiae* histones, and suggests that MBF may play a role, along with other mechanisms, in regulating histone expression in *S. pombe*.

The Wos2 cluster (Figure 9C) contains seven genes expressed in late S or very early G2. Expression of the genes in the cluster responds strongly to the two experiments that involve temperature shifts (*cdc25–22* synchrony and *cdc10-M17* block-release; note that control cDNA for simple cell cycle–arrest experiments was made from wild-type cells similarly shifted to high temperature). Motif searches found repeats of the sequence NGAAN, a typical heat shock response element. The cluster contains *wos2,* encoding a chaperone activator interacting with Hsp90; *SPACUNK4.16c,* important in trehalose synthesis (trehalose is a thermo-protectant); *SPBC16D10.08c,* encoding a chaperone similar to *S. cerevisiae* Hsp104; and *SPBC4F6.17c,* similar to *S. cerevisiae* *Hsp78,* a mitochondrial chaperone.

#### The early to mid G2 genes: The Ribosome biogenesis and Cdc2 clusters

**Figure 10:**
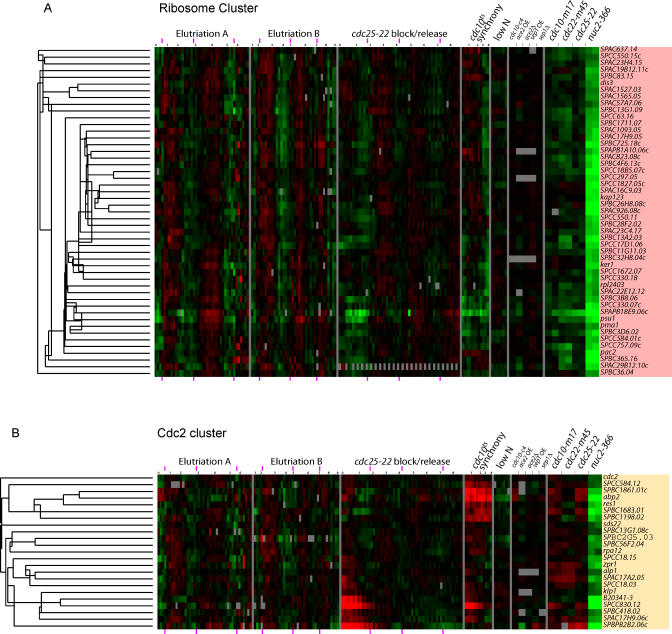
Early to Mid G2 Clusters (A) Ribosome biogenesis cluster and (B) Cdc2 cluster.

Although most of the strongly regulated genes peak near the G2/M transition, another large group of genes, 200 or more, peaks with a moderate amplitude at almost exactly the opposite side of the cell cycle, in early to mid G2 (Fig. 10). The expression of these genes does not respond to mutations in *cdc10, ace2,* or forkhead, and they are all strongly repressed at the *nuc2* block. Near the center of this set of 200-plus genes is a sub-cluster of genes that is somewhat more tightly co-regulated than the rest. We have designated these the “Ribosomal biogenesis” cluster (Figure 10A). These genes include*SPAC1527.03* (RNA-binding protein, LA-related), *SPAC57A7.06* (processome component, involved in rRNA processing), *SPBC13G1.09* (bystin family protein, associated with U3 and U14 snoRNAs, involved in rRNA processing); *SPCC16A11.02* (WD-repeat protein, processome component, involved in rRNA processing);*SPAC23C4.17* (tRNA methyltransferase of the NOL1/NOP2/sun family involved in methylation of cytidine to 5-methyl-cytidine [m5C] at several positions in different tRNAs);*SPBC11G11.03* (60S acidic ribosomal protein); *rpl2403* (60S ribosomal protein L24–3); *ker1* (interacts with RNA polymerase I); *SPAC1093.05* (DEAD/DEAH box RNA helicase involved in rRNA processing); *SPAC926.08c* (Brix domain RNA-binding protein involved in ribosome biogenesis and assembly); and many others.

Other genes in the ribosome biogenesis cluster are involved in nuclear/cytoplasmic import and export. These genes include: *nup61* (nucleoporin with a RanBp-binding domain), *kap123* (karyopherin), *SPCC550.11* (RanBP7/importin-beta/Cse1p family, RanGTP-binding protein involved in mRNA export), and *mep33* (mRNA export protein).

It is not clear why such genes would be cell cycle regulated. However, Mitchison and colleagues [37–41] have documented a cell cycle oscillation in the rate of growth and protein synthesis in *S. pombe*. In these studies, there seems to be an acceleration of protein synthesis, and a corresponding acceleration in cell growth rate, in mid G2. Furthermore, “NETO” (new end take off, the time when the new end begins to grow) occurs at about this time. The peak in expression of ribosome biogenesis genes we observe in early/mid G2 could lead to this slightly later peak of protein synthesis and growth rate. Sveiczer et al. [41] suggest that the acceleration in protein synthesis is the “sizer” that leads to commitment to division; in terms of our findings, the peak in transcription of the ribosome biosynthesis genes would be an important component of the sizer.

**Figure 11:**
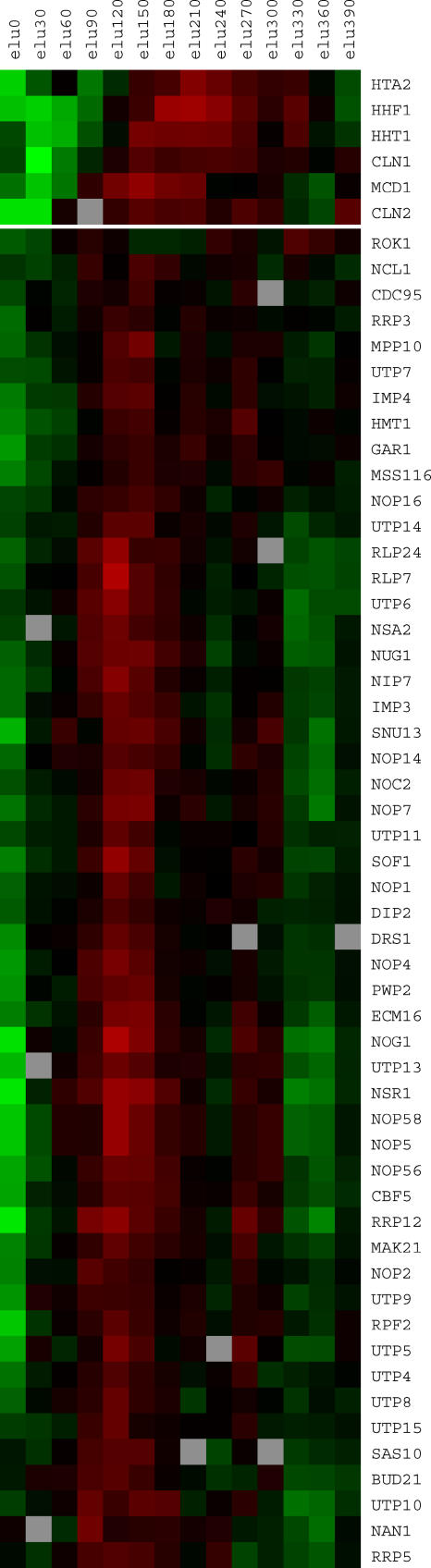
Oscillation of Ribosome Biogenesis Genes in *S. cerevisiae* One cell cycle of elutriation data is shown for 52 *S. cerevisiae* genes involved in ribosome biogenesis. The genes chosen for analysis were those listed [42,75] as involved in ribosome biogenesis. At the top of Figure 11 are three histone genes *(HTA2, HHF1,* and *HHT10)* known to peak in S, and three genes *(CLN1, CLN2,* and *MCD1)* known to peak in late G1. The raw data for this figure are taken from Spellman et al. [6]. In their experiment, cells were grown in ethanol medium, and then small G1 cells were isolated by elutriation and re-inoculated into ethanol medium. Samples were taken at intervals from 0 to 390 min, the duration of one cell cycle under these conditions.

We have recently found that many *S. cerevisiae* ribosome biogenesis genes are also cell cycle regulated (Figure 11). Expression peaks in G1, and so this peak could be important for the cell sizer, which in *S. cerevisiae* is in late G1. These genes also show a minor expression peak in early G2. The oscillation of these genes is seen in an elutriation experiment done in ethanol medium, but not in block-release experiments done in glucose medium. The reason for these different, experiment-specific results is unclear, but on the basis of the literature we believe that the oscillation may be under the control of cyclic AMP, and this cyclic AMP signaling does not occur in media with high glucose [42,43].

Surprisingly, we found no DNA sequence motifs associated with the promoters of the genes in the ribosomal biogenesis cluster.

As one moves out from the center of the ribosome biogenesis cluster, one encounters many other genes peaking in G2 phase. These are of diverse function, but one interesting example is the *pma1* gene, which encodes a proton pump. This pump is needed to maintain the proton gradient across the plasma membrane, affecting many processes, and so seems an unlikely candidate for a cell cycle–regulated gene. Nevertheless, it is cell cycle regulated both here and in *S. cerevisiae* [6]. The reason for the oscillation is unclear, but because Pma1 is an integral plasma membrane protein that must be inserted into the membrane at the time of synthesis, one possibility is that its synthesis matches the rate of plasma membrane production; in *S. cerevisiae,* this may reach a peak in G2, accounting for the peak in *PMA1* transcription. A similar explanation could hold true in *S. pombe*.

Adjacent to the Ribosome biogenesis cluster is a cluster of 23 genes we call the Cdc2 cluster (see Figure 10B). Like the ribosome biogenesis genes, these oscillate moderately with a peak in G2. Their regulation is distinguished from the ribosome biogenesis genes by the fact that they are differentially regulated after heat stress. Motif search programs found heat shock motifs (NGAAN) associated with many of these genes. The Cdc2 cluster contains several interesting cell cycle genes, including *cdc2* itself; *SPBC1861.01c* and *abp2*, which code for AT hook proteins thought to bind centromeric DNA and ARS DNA, respectively;*res1,* a key component of the MBF transcription factor; *sds22,* a protein phosphatase regulatory subunit known to be involved in the cell cycle; *ash2,* a member of the SET1 complex, and involved in lysine methylation of histone H3; *alp1,* a tubulin-specific chaperone; *SPCC18.03,* a putative transcriptional regulator; *pkl1,* a kinesin-like protein of the Kar3 family; and other genes. Other than the heat shock elements, no statistically significant motifs were associated with the promoters of these genes.

### Characterization of Cell Cycle–Regulated Promoters

Each cluster was searched for DNA sequence motifs. The most significant motifs are summarized in Table 1. However, the presence of these motifs in the upstream regions of the genes of a cluster says little about promoter structure. To investigate promoter structure in more detail, we used a program called SpikeChart (S. Pyne, B. Futcher, and S. Skiena, unpublished data) that finds and displays motifs in DNA sequences. SpikeChart uses a weight matrix to define a consensus motif, and it shows each occurrence of a motif as a spike of varying height depending on that motif's match to the consensus. For instance, a motif that matches the consensus motif exactly would be given a spike height of ten, whereas a motif with one or more mismatches to the consensus would be given a lower score, depending on the number and nature of the mismatches. (Weight matrices and scoring functions are shown in Table S2). SpikeChart can score many different kinds of motifs simultaneously, and can show the position of all scored motifs, so it is well suited to finding groups of motifs, whether they be of the same kind or different kinds. Initially, because we did not know where regulatory motifs might occur, SpikeChart was used to examine the first 200 base pairs (bp) of the open reading frame in question, and 2,000 bp upstream of the start codon (regardless of whether this region included the next open reading frame or not).

**Table 1:**
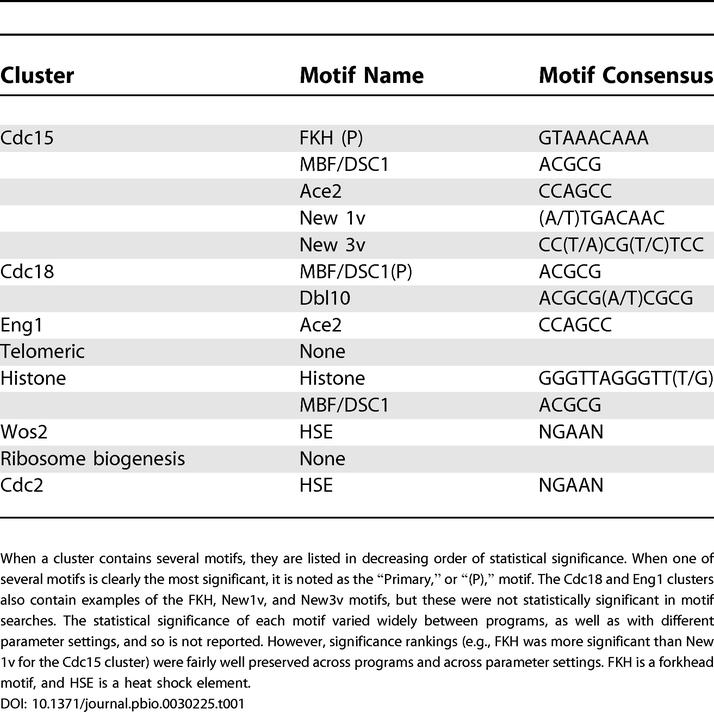
Clusters and Motifs

**Figure 12:**
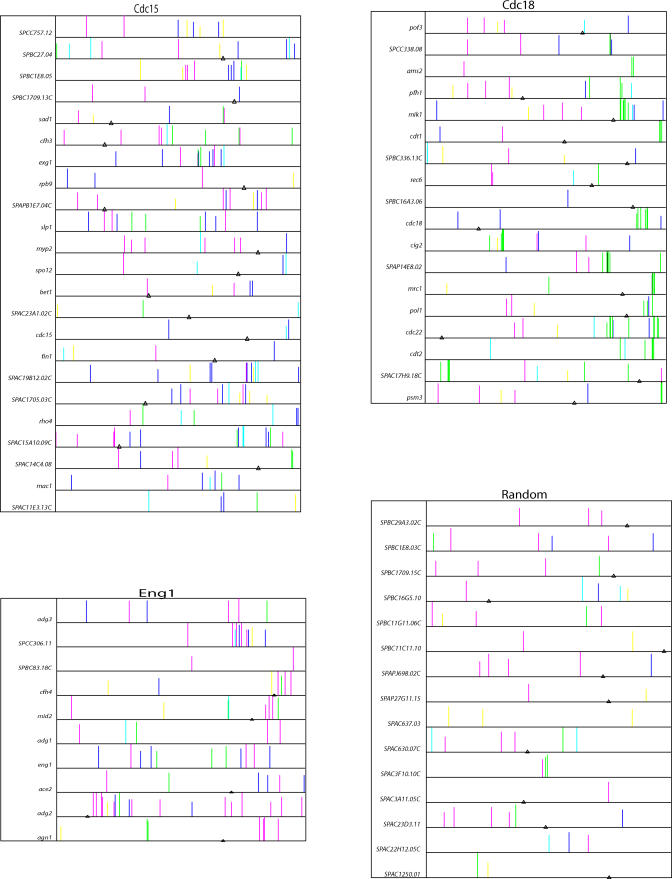
Distribution of Promoter Motifs A total of 23 genes from the core of the Cdc15 cluster (cdc15), the 18 genes from the Cdc18 cluster (cdc18), the nine genes from the Eng1 cluster, plus one similarly regulated gene (*SPBC83.18c*; eng1) and 15 randomly chosen genes (Random) had their promoters examined for six sequence motifs using SpikeChart. For each gene, the DNA sequence examined was the 2,000 bp immediately upstream of the Start codon; the Start codon is at the right edge of the figure, and the upstream 2,000 bp extend to the left. The beginning of the next upstream open reading frame is indicated by a triangle; for instance, for the *pof3* gene (Cdc18 cluster), the next open reading frame begins about 700 bp upstream of the *pof3* Start codon. For the Cdc18 cluster gene *ams2,* all 2,000 bp are intergenic. Consensus motifs are as follows: Dark Blue Fkh motif, TGTAAACAAA; Purple Ace2 motif, ACCAGCCT; Green MBF motif, GACGCGTC; Black Dbl10 motif, ACGCGACGCG; Light Blue/Aquamarine New 1v motif, TGACAAC; Yellow New 3v motif, (A/T)ACC(A/T)CG(T/C)(A/T)(C/A)C. Taller spikes indicate a better match to the consensus; the weight matrices, and the rules governing spike height, are given in Table S2. Dbl10 spikes (black) are obscured by overlapping MBF spikes (green), and so are hard to see. Only tall spikes (i.e., good matches to the consensus) are shown, so an acceptable motif may exist even when no spike is shown,

Groups of closely spaced, multiple motifs were usually visible, and these groups usually occurred in the upstream intergenic region (as opposed to within the open reading frame) (Figure 12). These groups of motifs were striking for their complexity. There were often four to ten motifs per group, and often the motifs were of several different kinds. The groups of motifs usually occupied about 400 bp of DNA. SpikeChart confirmed that the Cdc15 cluster was dominated by FKH motifs; the Cdc18 cluster was dominated by MBF and DBL10 motifs; and the Eng1 cluster was dominated by Ace2 motifs. However, SpikeChart showed that in addition to the predominant motif, the genes of all these clusters often had other motifs as well. In particular, for M phase genes, it was very common to have at least one FKH motif and at least one other kind of motif. There was a weak to moderate correlation between the number of motifs upstream of a gene and the amplitude of that gene's cell cycle oscillation (data not shown).

We did not notice any cases where the group of regulatory motifs was inside an open reading frame (either the downstream or upstream open reading frame).

In long (>1 kb) intergenic regions, the group of motifs usually occurred within 800 bp of the start codon, but this was not always true; a substantial minority of regulatory motif clusters occurred more than 800 bp upstream (but still within the intergenic region). Because the median *S. pombe* intergenic region is only 900 bp, we wondered whether the cell cycle genes might have unusually long promoters. We measured the length of upstream intergenic regions versus cell cycle rank in our list of all 5,000 genes. The most strongly regulated 200 genes had upstream intergenic regions of about 1,200-bp median length, versus a genome-wide median length of 900 bp. Thus, the more strongly cell cycle–regulated genes have longer than average upstream regions. We have noticed the same phenomenon with the cell cycle regulated genes of *S. cerevisiae* (S. Pyne, S. Skiena, and B. Futcher, unpublished data). The longer-than-average promoters found for cell cycle–regulated genes suggests that these promoters might be above average in complexity.

## Discussion

### How Many Cell Cycle–Regulated Genes Are There?

We have ranked *S. pombe* genes by the statistical significance of their oscillation, and we have discussed the most cyclic 750 genes. However, *p-*values (see Table S1) and other evidence (see Figure 6) suggest there are at least 2,000 genes with weak oscillations. This number fits well with the combined results of our study and the studies of Rustici et al. [7] and Peng et al. [8]. The three cell cycle lists of 750, 407, and 747 genes, respectively, implicate a total of 1,373 genes. Each study has uniquely identified some genes, but in general these are not just errors, because the vast majority of the uniquely identified genes show some cyclicity in one or both of the other studies even though they do not rise above the cutoff in those studies. Thus we feel the three groups of investigators are each fishing 400 to 750 genes out of a pool of about 2,000 detectably oscillating genes. The three groups are in excellent agreement with respect to the strongly oscillating genes, but then diverge with respect to the more weakly oscillating ones (see Figure 5).

However, at the same time, it seems unlikely that 2,000 genes would be directly involved in the cell cycle. There might be at least two kinds of reasons for the observed oscillations. First, an oscillation might be adaptive; i.e., there might be natural selection in favor of the oscillation. The DNA synthesis genes (e.g., *cdc18, pol1,* and *cdc22*) in the Cdc18 cluster are examples of genes in which it is easy to believe that the oscillation is adaptive. But second, some oscillations may be incidental. That is, there might be no selective advantage whatsoever to the oscillation, but instead the oscillation is a secondary or indirect effect. For example, chromatin condenses during mitosis. At least in multicellular eukaryotes, mitosis is associated with genome-wide repression of transcription. If there is a similar loss of transcription during mitosis in *S. pombe,* and if our microarray experiments are sufficiently sensitive, we will detect this decreased transcription as a cell cycle oscillation with a trough in mitosis for essentially all genes (preferentially the genes with a short mRNA half-life). But this cell cycle oscillation, though real, does not imply that the oscillation of any of these genes is beneficial; instead, it is a secondary consequence of mitotic repression and chromatin condensation, which presumably is beneficial. Incidental oscillation might also arise when two genes are adjacent to each other. One of the genes might oscillate for adaptive reasons, but the oscillation of this gene might carry over to adjacent genes, for which natural selection is perhaps indifferent to oscillation.

How can we distinguish adaptive from incidental oscillation? First, adaptive oscillations are likely to be large-amplitude oscillations, whereas incidental oscillations are likely to be small-amplitude oscillations. Our cutoff at 750 genes is a crude first screen to enrich for genes with adaptive oscillation. Second, one should consider the total oscillation of the gene's final activity. That is, the oscillation of a gene's transcript might be small. But if one finds that the same gene also has an oscillation in protein stability (e.g., because of regulated proteolysis), and also an oscillation in enzyme activity (e.g., because of phosphorylation), this suggests that the oscillation is adaptively significant. For example, in *S. pombe,* the cyclin transcripts oscillate only modestly, and yet the oscillation of the final product (Cdc2 protein kinase activity) is large. The modest oscillation of the transcript contributes in a significant, multiplicative way to the overall oscillation, and is undoubtedly adaptive. Third, one should consider co-regulated genes and the mode of regulation. If a gene is a member of a small cluster of genes, and the genes have related functions and are regulated by a specific cell cycle transcription factor, then the oscillation is almost certainly adaptive. But if the gene is co-regulated with hundreds of other genes all with very small oscillations, and there is no common function to the genes and no known cell cycle transcription factor, then the oscillation of the whole set of genes may be secondary to some effect such as chromatin condensation. Fourth, one should consider the chromosomal location. Genes adjacent to adaptively regulated genes could oscillate passively. In particular, genes in regions of special chromatin structures (e.g., near telomeres, centromeres, and silenced regions) could oscillate as a secondary consequence of cell cycle changes in the special chromatin structure.

In summary, we feel that a very large number of *S. pombe* genes, 2,000 or more, have at least very small cell cycle oscillations. But it is possible that in many cases this oscillation may be incidental and that only a smaller but unknown number oscillate for adaptive reasons. Sorting adaptive from incidental oscillations will require additional experiments.

### Two Genome-Wide Waves of Transcription

There were two large waves of transcription, one peaking in early/mid G2, and the other peaking in late G2 or M (see Figure 6). The early/mid G2 wave contains hundreds of genes, including many genes for ribosome biogenesis. Interestingly, Mitchison and co-workers [41] have documented a cell cycle oscillation in protein synthesis, which peaks in mid G2, and may help trigger commitment to cell division. We believe that the early/mid G2 peak in ribosome biogenesis genes may lead to this slightly later peak in protein synthesis.

One property of these early/mid G2 genes is that they are deeply repressed at the *nuc2* block in mitosis (see Figure 10). This is reminiscent of mitotic repression, a phenomenon observed in multicellular eukaryotes in which the majority of transcription (Pol I, Pol II, and Pol III) is repressed during mitosis [44–52]. Repression is especially well established for Pol I and Pol III polymerases, which are needed for transcription of ribosomal RNA and other RNAs required for protein synthesis. It is thought that highly active transcription may interfere with chromosome condensation, and so transcription is repressed to allow condensation.

A related observation is that in the 1970s and 1980s, metabolic labeling studies were done on synchronized cultures of *S. pombe*. These studies found “steps” of incorporation of labeled uridine into RNA (mostly ribosomal RNA) as a function of cell cycle phase. Around mitosis, incorporation was poor, then after mitosis, the rate of incorporation increased, and then flattened out again at the next mitosis, then increased, and so on. The interpretations of this step-like, cell cycle–regulated uridine incorporation were varied, and the subject disappeared from the literature without resolution [53–56].

Putting these observations together, we speculate that *S. pombe,* too, may have some degree of mitotic repression, perhaps important for chromosome condensation. Pol I accounts for the vast majority of the transcription in the cell. Mitotic repression of Pol I transcription of the ribosomal RNA genes would account for the pause in uridine incorporation seen in mitosis in the metabolic labeling studies. But if ribosomal RNA is not transcribed in M, and given that the components of the ribosome are tightly coordinated in their production, then genes for ribosomal proteins (as seen by Peng et al. [8]), and genes for ribosome biogenesis, might also be repressed in M. Repression in M would account for the oscillation of the ribosome biogenesis cluster and its repression at a *nuc2* arrest. Finally, if the ribosome biogenesis genes cluster together because they are subject to mitotic repression, this might explain why the cluster does not contain any characteristic 5′ motifs: Mitotic repression might not work through a particular upstream site-specific transcription factor. Indeed, in *S. cerevisiae,* ribosome biogenesis transcripts are controlled in part at the level of mRNA stability [57]. Thus, we suggest that *S. pombe* may have a form of mitotic repression and that this repression in mitosis may account for the oscillation of the ribosome biogenesis genes and other genes peaking in early/mid G2 phase and troughing in M.

The second large wave of gene expression peaks in late G2 and in M. This wave includes the Cdc15 cluster (which has many genes for mitosis), the Cdc18 cluster (DNA replication), and the Eng1 cluster (cell separation). There are many important cell cycle events in M and S, and these two phases are close together in rapidly-growing *S. pombe*. The many genes peaking in late G2 and M may simply represent the cell's efforts to prepare for the many activities of M and S. It will be of interest to see what happens to the timing of the Cdc18 cluster (DNA synthesis genes) in slowly growing cells with a long G1: Will they still be transcribed in mitosis, or will they now be transcribed in late G1?

If mitotic repression does exist in *S. pombe,* how is it that the Cdc15, Cdc18, and Eng1 clusters peak in M phase? Baum et al. [58] have used nuclear run-on to show that *cdc18* and some other members of the cdc18 cluster can be actively transcribed in mitosis at a time when histone H1 kinase activity is high and chromatin is presumably condensed. Our own results agree that essentially all the genes of the Cdc15, Cdc18, and Eng1 clusters are highly expressed at a *nuc2* arrest, a time at which histone H1 kinase activity is high, and chromatin should be condensed. Our elutriation data suggest that in normal cells, the peak of expression of genes in the Cdc15 and the Cdc18 cluster is almost simultaneous with mitosis (see Figures 6 and 8). The genes of these clusters may be specialized for transcription in mitosis. Interestingly, the Cdc15 cluster genes have binding sites for a forkhead transcription factor. Forkhead transcription factors have a “winged-helix” fold, a structure they share with histone H1. Like histone H1, forkhead proteins may be capable of binding to linker DNA in between nucleosomes, and seem to be capable of binding even to chromatin that is relatively condensed [59–62]. That is, perhaps forkhead is an enabler of transcription for genes in condensed chromatin, and so is particularly suitable for driving expression of genes in mitosis. The Cdc18 cluster depends on the MBF factor, and the MBF/SBF/E2F family of DNA-binding proteins also has a winged helix fold [63]. Finally, the Ace2/Swi5 family of transcription factors has been associated with the recruitment of chromatin remodeling enzymes and histone acetylases [64]. Even in mammals, which clearly do have mitotic repression, there are mitotic genes strongly expressed during mitosis [65]. Interestingly, at least some of these genes are thought to be regulated by forkhead transcription factors [66].

The more moderately expressed genes in the G2/M wave (i.e., genes not in the Cdc15, Cdc18, or Eng1 clusters) tend to be expressed in late G2 rather than in M (see Figure 6C). Thus these genes may be subject to mitotic repression. Perhaps a large number of genes are expressed in late G2 because it the last chance to be expressed before M, a relatively inopportune time for transcription.

### Comparison of Cell Cycle Genes in *S. pombe* and *S. cerevisiae*

Of our top 200 ranked cell cycle–regulated genes, 72 (36%) had *S. cerevisiae* homologs that cycled, 68 had *S. cerevisiae* homologs that did not cycle significantly, and 60 did not have clear *S. cerevisiae* homologs. (A detailed comparison of the top 200 *S. pombe* genes and their *S. cerevisiae* homologs is available as Table S3).

Genes involved in core cell cycle processes such as DNA synthesis and mitosis were especially likely to cycle in both organisms. On the other hand, genes involved in budding (in *S. cerevisiae*) or fission (in *S. pombe*), or in cell wall carbohydrate metabolism, generally did not cycle in both organisms for the obvious reasons that the mechanism of cell separation, and the nature of the carbohydrates in the cell wall, are not conserved between the two yeasts.

There are many individual cases where a process is cell cycle–regulated in both organisms, but either the level of regulation (i.e., transcriptional or post-transcriptional) or the identity of the gene regulated varies between the two yeasts. One example is the activity of the cdc2/Cdc28 protein kinase. In *S. cerevisiae,* most of the cyclins are very strongly regulated at the transcriptional level (e.g., *CLN1,* *CLN2, CLB5, CLB6, CLB1,* and *CLB2*), but in *S. pombe,* the equivalent cyclins are only weakly or moderately regulated at the transcriptional level. Possibly compensating for this relatively weak transcriptional regulation, *S. pombe* has very strong post-translational regulation of Cdc2 kinase activity via Wee1/Mik1 inhibitory tyrosine phosphorylation of Cdc2, whereas the homologous system is relatively weak in *S. cerevisiae*. That is, both yeasts strongly regulate cdc2/Cdc28 activity through the cycle, but emphasize different mechanisms. A second example is provided by the gene products of *dut1 (SPAC644.05c)* and *ung1*. These proteins both work to exclude uracil from DNA, but by independent mechanisms. The Dut1 protein hydrolyses dUTP, whereas the Ung1 protein removes uracil from DNA by cleaving the glycosidic bond. In *S. cerevisiae,* *dut1* is very weakly cell cycle regulated, whereas *ung1* is moderately regulated. In *S. pombe,* *dut1 (SPAC644.05c)* is very strongly regulated, whereas *ung1* appears not to be regulated at all. Thus both yeasts use cell cycle transcriptional control to exclude uracil from DNA, but the emphasis is on different genes.

### Regulatory Networks and the Late G2 Bump

In *S. cerevisiae,* there is a regulatory network governing the transcription of cell cycle genes. This network is organized as a circular cascade, such that transcriptional and post-transcriptional changes occurring during one part of the cycle seem to promote changes in the next part of the cycle, and so on around a circle [67–69]. In principle, *S. pombe* must also have a circular cascade of some kind to make the cell cycle repeat. However, fewer cell cycle regulatory mechanisms have been described in *S. pombe* than in *S. cerevisiae,* and so the wiring of the putative cascade is still unclear. In particular, it is unclear how extensive a role is played by transcriptional control.

Moreover, in *S. cerevisiae,* genes displaying large-amplitude cell cycle changes are distributed throughout the cycle [6], consistent with the idea that transcriptional control contributes significantly to all phases of the cascade [67]. However, in *S. pombe,* most large-amplitude genes are expressed in a window near the G2/M transition, whereas genes of moderate and low amplitudes are distributed throughout the cycle. This concentration of large-amplitude genes near M may suggest that transcriptional control is most important for only some portions of the cascade.

Within the G2/M window of high-amplitude transcriptional regulation, one can discern what may be part of the regulatory wiring diagram. The transcription factor gene *fkh2* peaks in the earliest part of the late G2 window. Over 100 other genes in this window, including *fkh2* itself, have FKH binding sites, so the up-regulation of *fkh2* may contribute to this large wave of gene expression.

One of the critical targets of the Fkh transcription factor may be the gene for the Ace2 transcription factor. The *ace2* promoter has multiple sites for Fkh binding. The *ace2* promoter also has one site for Ace2, so, like the *fkh2* gene, *ace2* may be autoregulatory. The Ace2 transcription factor then induces a cluster of genes involved in cell separation and cell wall metabolism. Interestingly, a forkhead transcription factor is involved in turning on the *ACE2* gene in *S. cerevisiae,* so this particular part of the cell cycle wiring diagram appears to be conserved in the two species.

Three of the major cell cycle transcription factors in *S. cerevisiae,* MBF/SBF, Fkh, and Ace2/Swi5, have homologous cell cycle transcription factors in *S. pombe*. The major exception is Mcm1, a MADS-box transcription factor. In *S. cerevisiae,* there are two paralogs of this gene, *MCM1,* and *ARG80*. Mcm1 is a transcription factor for cell cycle genes and mating genes, whereas Arg80 controls various metabolic processes. The best *S. pombe* orthologs are Map1 and Mbx1 [19]. There was no noticeable enrichment of an Mcm1-like binding motif in front of any cluster of cell cycle–regulated genes; i.e., there was no evidence for a binding site for Map1 or Mbx1.

In multicellular animals, the major well-characterized cell cycle transcription factor(s) are those of the E2F/DP family [70,71]. These typically control a cluster of genes expressed in late G1, and the genes are involved in DNA replication and commitment to the cell cycle. Functionally, the genes controlled by E2F/DP in animals are similar to the genes controlled by MBF in the two yeasts. E2F and DP proteins are not very similar in sequence to the proteins found in MBF, but it is also true than various E2F and DP proteins are not very similar to each other, though they are clearly related. E2F and DP recognize binding sites with a CGCG core, as does MBF. Furthermore, the DNA-binding domain of E2F/DP factors consists of a winged-helix fold [72], as do the DNA-binding domains of Swi4 and Mbp1 (components of *S. cerevisiae* SBF and MBF, respectively) [63,72]. Thus, despite the overall sequence dissimilarity, it is possible that MBF in the yeasts, and E2F/DP in animals, are cell cycle transcription factors that are related by descent and which have always controlled the cell cycle expression of genes involved in DNA replication.

## Materials and Methods

### Microarrays

Microarrays were made by spotting unmodified, double-stranded PCR products onto glass slides coated with aminopropylsilane (Erie Scientific). Spotting was done using a robot of the DeRisi design (http://cmgm.stanford.edu/pbrown/mguide/) and ArrayMaker2 software (http://derisilab.ucsf.edu/arraymaker.shtml). PCR primers were designed using Primer3 (Whitehead Institute, Cambridge, Massachusetts, United States) and a shell script. Primers were designed against approximately 5,000 open reading frames and RNAs (excluding pseudogenes) as annotated by the Sanger Centre (http://www.sanger.ac.uk/Projects/S_pombe/DNA_download.shtml). In general, PCR primer pairs were designed to give products 500 to 1,000 bp in length, because the yield of the PCR reaction decreased for products longer than 1,000 bp. When the PCR product was small compared to the length of the gene, it was usually chosen from the 3′ region of the gene, so as to maximize representation in poly dT-primed cDNA synthesis. PCR products were amplified from genomic *S. pombe* DNA, and so in some cases the final product included introns, but the design parameters maximize contiguous exonic sequence. A fuller description of the microarrays will be published elsewhere. A full description of the primer pairs, and hence the features on the microarrays, can be found at http://www.redgreengene.com.

### Cell cycle synchronizations

Two methods of cell cycle synchronization were used, elutriation and a *cdc25–22* block and release. Two independent elutriation experiments were carried out. For elutriations, 8 l of h-972 cells (wild-type) were grown in YES (autoclaved, elutriation B or filter-sterilized, elutriation A) to early log phase (OD_600_ = 0.4) at room temperature (25 °C). Cells were harvested by centrifugation, resuspended in approximately 100-ml YES, and sonicated, all at room temperature. For elutriation B, approximately half of the cell volume was reserved for the reference cDNA preparation. For elutriation A, the reference cDNA was prepared independently and the entire sample was used for elutriation. Cells were loaded into a Beckman elutriator rotor containing two 40-ml elutriation chambers connected in series. When two chambers are used in series, the bulk of the cells remain in the first chamber, but the smallest cells flow into the second chamber, and then, at higher pump speeds, some of these flow out of the elutriator for collection. This arrangement provides both high capacity and high resolution. The elutriator was used at 1,800 rpm at room temperature. After every increase in pump speed, a fraction of about 150 ml was collected, containing about 5 × 10^8^ cells (elutriation B) or 3 × 10^9^ cells (elutriation A). These were diluted to OD_600_ 0.2–0.05 (greater dilution for samples harvested at late times) with conditioned (elutriation B) or fresh filter-sterilized (elutriation A) medium, and then sampled with time.

An entire three cell cycle time course was obtained from five elutriator fractions (elutriation B) or two fractions (elutriation A). We used adjacent fractions containing no (< 0.5%) septated cells; the elutriator fraction with the largest cells (i.e., the last fraction collected) was used first, then the elutriator fraction with the next largest cells, and finally the elutriator fraction with the smallest cells (i.e., the first fraction collected). In general, the fractions were “overlapped,” i.e., the last sample from one fraction and the first sample from the next fraction were collected at the same time. “Overlapped” fractions, though collected at the same time, were deemed to have been collected at slightly different times; the number of minutes by which overlapped fractions were offset was determined by the offset, in minutes, of the septation indicies for the two fractions. That is, for any pair of overlapped fractions, the smaller cells were deemed to have been collected earlier, by a time determined from the offset of the septation indicies of the two fractions. Note that elutration A used only two fractions, and so there was only one overlap. Samples were taken about 10 min. (elutriation A) or 15 min (elutriation B) apart; exact sampling times are given in the Treeview files 1, 2, and 3 (Dataset S1) and at http://www.redgreengene.com.

Cells (10^8^ cells/sample) were harvested by centrifugation at 4 °C and washed with ice-cold water, snap frozen, and stored at −70 °C. For elutriation A, an equal volume of ice was added to the cell culture during harvest (harvest with ice). The reference sample for hybridizations was sonicated cells prior to elutriation (elutriation B), or h-972 grown to OD600 0.2 in filtered YES at 25 °C (elutriation A). Septation index was monitored by phase contrast microscopy of live cells during each experiment. In addition, frozen cell pellets were thawed and stained with DAPI and calcofluor to monitor anaphase (“binucleates”) and septation for elutriation A. Cells were scored as “binucleates” if two nuclei were visible, but there was no septum.

For the *cdc25–22* block release, the prototrophic strain JLP1164 h+ *cdc25–22* was grown in filtered YES at 25 °C to OD_600_ = 0.4 and then used to inoculate 4 × 500 ml filtered YES to an OD_600_ of 0.1 (flask 1), 0.08 (flask 2), 0.07 (flask 3), and 0.05 (flask 4). Cells were shifted to a water bath at 36.5 °C for 4 h to arrest them in G2 (time = 0 h) and then shifted back to 25 °C rapidly in an ice-water bath (26 °C was achieved in approximately 5 min; cultures did not cool below 25 °C). Samples were taken 10 min apart and harvested with ice as described above. The reference sample for hybridizations was JLP1164 h + *cdc25–22* grown at 25 °C to OD_600_ = 0.2 in filtered YES. Septation index was monitored by phase contrast microscopy.

### Other microarray experiments

To examine cells released synchronously from a *cdc10* arrest, 8 l of strain JLP1166 h− *cdc10-M17* was grown at 25 °C to OD_600_ = 0.5 in filtered YES, and then harvested and elutriated to obtain a fraction of G2 cells. These were diluted to 10^6^ cells/ml, shifted to 36.5 °C for 3 h 15 min, rapidly cooled to 25 °C as described above (time = 0), and then sampled with time. Cells were harvested with ice. Samples were also collected and analyzed by flow cytometry to monitor DNA replication. The reference sample for hybridizations was JLP1166 *h− cdc10-M17* grown to OD_600_ 0.2 in YES at 25 °C.

To examine cells grown in low nitrogen, wild-type h-972 was grown in EMM lacking NH_4_ and supplemented with 20 mM phenylalanine (EMM-phe) to provide a limiting nitrogen source to expand the G1 window [73]. 8 l of cells were grown at 25 °C to OD_600_ = 0.4, collected by centrifugation at 4 °C and kept on ice and sonicated on ice. Approximately half of the total cell volume (125 ml, total 2 × 10^8^ cells) was reserved for reference cDNA synthesis and the remainder was elutriated at 4 °C to fractionate the culture into 21 fractions ranging from small cells (50% G1) and then medium cells (G2) and finally to long, septated cells. Fractions were harvested immediately by centrifugation at 4 °C. Fraction assignments were confirmed by flow cytometry analysis and high-quality hybridizations were obtained with fractions 2, 3, 5, 7, 10, 13, and 16.

To examine cells arrested at the *cdc10, cdc22, cdc25,* and *nuc2* block points, four strains carrying these cell cycle mutants *(cdc22-M45, nuc2–663, cdc25–22,* and *cdc10-M17)* and a wild-type reference control were grown to OD 0.05–0.08 in YES at 25 °C and shifted to 36.5 °C. After 4 h of arrest at this restrictive temperature, a sample was taken for microarray analysis. For each strain, the experiment was repeated with an independent single colony. Figures 6, 7, and 9 show results (in different columns) from both single colonies. Strains used were wild-type PR109 *h− leu1–32 ura4-*D18 (obtained from P. Russell), and the cell cycle mutants (F84) OM591 *h− cdc22-M45* (P. Russell), (F58) PR580 *h− leu1–32 nuc2–663* (P. Russell), JLP1165 *h+ cdc25–22* (this study), and JLP1166 *h− cdc10-M17* (this study).

### Microarray hybridization and processing

Cell samples for RNA isolation were rapidly cooled by addition of an equal volume of ice (except for elutriation B in which samples were placed on ice) and then collected by centrifugation at 4,000 rpm. (3,300 × g) at 4 °C for 3 min. Pellets were washed twice in ice-cold dH20, frozen in liquid nitrogen, and stored at −70 °C. Total RNA was isolated using RiboPure Yeast (Ambion, Austin, Texas, United States) according to the manufacturer's instructions (elutriation A samples) or hot phenol essentially as described [7] (http://www.sanger.ac.uk/PostGenomics/S_pombe/docs/rnaextraction_website.pdf with slight modifications according to the detailed protocols at http://www.redgreengene.com). Isolated RNA was further purified by RNAeasy cleanup columns (Qiagen, Valencia, California, United States) and quantitated by absorption spectroscopy.

Microarray probes were prepared in two steps. First, cDNA was synthesized incorporating aminoallyl-dUTP (aadUTP). Purified aadUTP cDNA was then coupled with Cy3 or Cy5 fluorescent dyes according to protocols from the Institute for Genomic Research (http://www.tigr.org/tdb/microarray/protocolsTIGR.shtml) with slight changes (http://www.redgreengene.com) as follows: 20–25 μg of total RNA was used for cDNA synthesis with 4 μg of oligo-dT primer (not random hexamers), and reactions contained 300 μM aminoallyl-dUTP with 200 μM dTTP. RNA was destroyed using RNase instead of NaOH, and reactions were purified with a Qiagen PCR purification kit. Dye incorporation was determined by absorption spectra and was typically one fluor/20–30 nucleotides.

For hybridizations, cDNA with 50 pmol Cy3 plus reference cDNA with 50 pmol Cy5 was included in a 24 μl total hybridization solution (25% v/v formamide, 5× SSC, 0.1% SDS, and 100 μg/ml of sonicated salmon sperm DNA). Hybridizations were performed under 22 × 25 mm lifter cover slips (Erie Scientific, Portsmouth, New Hampshire, United States) at 50 °C in a humidified chamber for 16–20 h. Hybridized arrays were washed by gently shaking as follows: twice briefly with 2× SSC/0.1% SDS (50 °C), twice for 10 min with 2× SSC/0.1% SDS (50 °C), and four times briefly with 0.1× SSC at room temperature. Arrays were dried by centrifugation.

Arrays were scanned using an Axon 4000B scanner, controlled by GenePix Pro 5.1 software with a pixel size of 5 μm and two-pass sequential line averaging. Laser power was set to 100%, and PMT gains were subjectively adjusted during prescan to maximize effective dynamic range and to limit image saturation. Lossless image files were stored for later analysis.

### Data extraction and storage

To extract data from microarray scans, previously stored image files representing all hybridizations were analyzed in parallel. Spot size, location, and quality were determined automatically by GenePix Pro algorithms. Dynamic spot resizing between 60 and 150 μm diameter was permitted based upon image examination and prior optimization. Misidentification of spot locations was corrected by manual adjustment of the map prior to automatic sizing and shifting. Only in cases of gross hybridization defect were spots/regions manually moved/resized or flags modified to “bad,” permitting consistent spot calling. Following spot location, parameters and values for each spot were calculated by GenePix Pro and exported. No normalization was applied within GenePix Pro.

Raw data and images exported from GenePix Pro were used to populate a local installation of the Longhorn Array Database (Peter Killion, University of Texas at Austin, http://www.longhornarraydatabase.org, an SQL database based upon the Stanford Microarray Database (http://yeastgenome.org, Stanford University). Initial data normalization was performed at the time of population. Briefly, spots were categorized as “pombe” or “other.” Pombe spots were further categorized into “normalization” (no bad, missing, absent, or not-found flags) or “non-normalization” (bad, missing, absent, or not-found flags). Only normalization spots were further considered for the normalization calculation. Finally, spots with greater than 5% saturation in either image channel were discarded from this group. The mean log_2_ ratio of the median net intensities (*R*_m_, foreground pixel − median of the local background) was calculated. This “normalization factor” represented the distance from a red/green ratio of one, and was used as a scalar modifier for the ratios of all spots in the hybridization; i.e., though only spots meeting a stringent “good” criterion were used to determine the normalization value, this value was subsequently applied to all spots, good or not. During retrieval of data, several further criteria were used to ensure high-quality data in downstream analysis. Only spots with non-negative flag values were retrieved (not bad, missing, absent, or not-found), and only spots with a regression correlation of pixel ratios (a metric of internal spot consistency) greater than 0.6 were used. Spot values were averaged (mean) when multiple independent spots representing a single PCR product were present as internal controls or otherwise. When analyzing multiple hybridizations, such as during time-course analysis, iterative gene and array centering was performed. Briefly, within an array of genes × arrays, the mean log_2_ ratio of medians (*R*_m_) was calculated and subtracted from each log_2_ *R*_m_, first along the gene axis, then along the array axis, until subsequent iterations varied by < 0.001%.

### Normalization

There are some special red/green normalization issues relevant to the genome-wide waves of expression (see Figure 6). In general, fluorescence from mRNA from synchronous cells was normalized to total fluorescence from mRNA from asynchronous cells. If all mRNAs were equally reduced in abundance during mitosis (e.g., because of genome-wide mitotic repression), this normalization would obscure the effect. However, despite normalization, any such repression would still be somewhat visible as a relative loss of unstable mRNAs versus stable mRNAs. We presume that if the trough of peaks of expression in M/G1/S seen in Figure 6 is indeed partially due to mitotic repression of transcription, then the individual genes troughing at this time are genes that produce unstable mRNAs, which are then noticeably repressed despite the red/green normalization because they are reduced relative to stable mRNAs. This argument suggests that stable mRNAs should tend to peak in the M/G1 interval. Indeed, some genes do peak at this time. These, however, would be fewer in number than the genes that fail to peak, because, in general, cells tend to express a small number of stable mRNAs to very high levels, comprising the bulk of mRNA, and a large number of unstable mRNAs to low levels [74].

A second normalization issue is that the oscillation of the strongly regulated genes would have an effect, via normalization, on the apparent expression of non-oscillating genes (i.e., genes that do oscillate would produce an artifactual, complementary oscillation in non-oscillating genes, via normalization). To side-step this artifact, pixel intensity data for the bottom 4,000 genes were extracted from the microarray data before the red/green normalization step, and then normalized and analyzed after extraction, so that the oscillation of the 1,000 most strongly cyclic genes would not interfere with normalization of the least cyclic genes.

### Cluster analysis

For cluster analysis, array- and gene- centered log_2_ *R*_m_ data were hierarchically clustered along the gene axis by the agglomerative algorithm of Eisen et al. [10]. Data were visually presented using JavaTreeView (http://jtreeview.sf.net and http://jtreeview.sourceforge.net/manual.html). Separation of the total dendrogram into subordinate clusters was performed subjectively.

### Motif analysis

To find DNA sequence motifs, nucleotides extending from −1 to the edge of the most 3′ proximal gene (stop or ATG, depending on orientation) with a maximum length of 12,000 bp were extracted genewise for each cluster and used as a target set for motif searching. Three different motif search programs were used: MEME, AlignAce, and SPEXS.

MEME (Multiple EM for Motif Elicitation) [15] was used to find motifs between five and nine nucleotides long present in any number of copies on either strand, weighted to find 1/3*n* to 3*n* total sites in the target set of n sequences. Parameters were set as follows: $ meme (sequence.name) –dna –minsites (*n*/3) –maxsites (n*3) –mod anr –minw 5 –maxw 9 –revcomp –nmotifs 10 –evt 0.1 –bfile (fifth order Markov model). (Other parameters were also tried in additional searches.) The top ten motifs exceeding an *E*-value of 0.1 were generated using a background set consisting of the fifth order Markov model representing possible nucleotide pentuplets in all *S. pombe* upstream regions.

AlignACE [12] uses a Gibbs sampling algorithm. Again, all *S. pombe* upstream regions were used as a background set.

SPEXS (Sequence Pattern EXhaustive Search) [14], a word-search enumeration algorithm, was also used. Relative frequency of 1- to 9-mers was calculated, and compared between the target set and all *S. pombe* upstream sequences.

### Identification of oscillating transcripts

In general, identification of oscillating transcripts requires a method for finding oscillations in each experiment, and then a method for combining the results from different experiments. Here, we have used Fourier analysis to identify oscillating genes. A *p-*value for the hypothesis of oscillation was then established using Monte Carlo simulations on shuffled data. The *p-*values for different experiments were then combined using known statistical properties of the *p-*value. Finally, the *p-*values for each gene were ranked. Although we have ranked the *p-*values, these *p-*values are nevertheless closely correlated with the amplitude of the oscillation.

For each time series of observations in a single time course (e.g., a three cell cycle elutriation experiment), we calculated the Fourier sums *A* and *B* over the range of times, *t,* in the experiment:



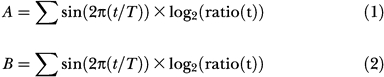



Here, *t* is the time in minutes at which the sample was taken (where the beginning of sampling is zero time); *T* is the cell cycle period, i.e., the time in minutes required for a complete cell cycle; and ratio(*t*) is the ratio of experimental to control signal at time *t.*

We considered these two sums as a vector *C* = (*A,B*), and then calculated the magnitude of the vector, *D*_O_ = square root of (*A* ^2^ + *B* ^2^). This magnitude, *D*_O_, is our basic Fourier measure of whether a transcript oscillates. Note that there is no need to calculate phase.

However, random noise would generate some value of *D* greater than zero, and genes whose transcripts are relatively variable in abundance could generate relatively large values of *D,* even if these variations had no connection to the cell cycle. Therefore, as a second step, we randomly shuffled the series of observations for each gene in question, and calculated a new magnitude, *D*_R_, for the randomized series. This randomization was repeated 1,000 times, generating 1,000 values of *D*_R_. These represent the distribution of *D* for each gene, given that gene's actual variance in gene expression. Finally, we compared the original value of *D*_O_ from the unshuffled data to the distribution of *D* from the shuffled data, found how many standard deviations *D*_O_ is from the mean of the distribution, and in this way calculated a *z* score for *D*_O_.

This procedure was repeated for each gene and for each experiment. Thus, for each gene, there were three *z* scores, one per experiment (two elutriation experiments and the *cdc25* block-release experiment). These three *z* scores were then combined by the method of Stouffer, yielding a single *p-*value for each gene. Genes were then ranked by *p-*value with the lowest *p-*value at the top of the list. In practice, a large amplitude of oscillation contributes tremendously to a low *p-*value, so the upper portion of the *p-*value list is almost exclusively occupied by genes with high-amplitude oscillations

### Gene database

In general, we have used the information in the GeneDB database (http://www.genedb.org/genedb/pombe/index.jsp) to describe the various genes studied; when a fact is given in the text about some gene but no reference is given, the information comes from GeneDB. When the primary literature has been consulted directly, the reference for the primary literature is given.

## Supporting Information

Dataset S1 Treeview Files Datasets appropriate for Treeview (see below) are provided as a tar.gz file. Upon opening, this tar.gz file will create a folder containing the three supplementary dataset files S1, S2, and S3 (pombecellcycle.cdt, pombecellcycle.gtr, and pombecellcycle.jtv) along with an additional “treeview_configuration.txt” file detailing the configuration of Treeview to access Sanger GeneDB for *S. pombe.*
These cdt, gtr, and jtv files can then be used to view clusters of the top 750 genes in a convenient and searchable way using Treeview, an open-source cluster visualization package available for many different platforms (http://jtreeview.sourceforge.net). Launch Java Treeview and open file pombecellcycle.cdt. The two supporting files will be accessed automatically (given that they are in the same folder). The pombecellcycle.jtv file is not required and only provides configuration settings. Java Treeview can be instructed to link directly to GeneDB (or any other database) so that a user can quickly check information on any given gene in a cluster of interest. To configure a copy of Java Treeview to link to GeneDB, do the following. First, in “Settings” go to “gene URL presets” and change one of the presets to name GeneDB and template http://www.genedb.org/genedb/Search?organism=pombe&name=HEADER&isid=true and choose this as default. Second, go to Settings “URL settings” and select gene; check that your new template is selected and that the “UID” setting is chosen. Now when an individual gene in a cluster is selected, the GeneDB Web page for that gene should open automatically. Documentation for Java Treeview is available at http://jtreeview.sourceforge.net/manual.html. The cdt file contains log ratios for each gene at each timepoint, and the timepoint names also contain information on septation index (SI) for all three synchrony experiments and binucleates for elutriation A. The form of the header for timepoint names is: [experiment]_[time]min_[%]SI_[%]BN. SI indicates Septation Index (i.e., percent septation), assayed by calcofluor for “ElutA,” phase contrast for “ElutB,” and *cdc25*. “BN” indicates the binucleate “anaphase index” as measured by DAPI staining, applicable only to ElutA. These headers are displayed in the Zoom window of Java Treeview.
(210 KB ZIP).

Figure S1 Oscillation of *cdc18*
The oscillation of the *cdc18* transcript through two cell cycles in elutriation A is plotted as a histogram (right *y*-axis). Also shown are the binucleate (blue triangles) and septation indices (cyan squares) (left *y*-axis).
(165 KB EPS).

Table S1 Cell Cycle Parameters of *S. pombe* Genes Data is presented for all 4,988 genes analyzed. Column headings are as follows: “SUID” is the systematic name. “Common-name” is the common name. “Rank” is the rank in our *p-*value list, from smallest *p-*values (i.e., most significant genes) to largest *p-*values. A *p-*value of “0” means a *p-*value of less than 10^−16^. “Desc” is a one-line description of the gene from GeneDB. “Cluster” is the cluster to which the gene belongs, if any. “ElutA_Phase” is the phase angle of the gene calculated from elutriation A. Phase angles range from 0° to 360° (i.e., around a circle). The phase angles of landmark events are as follows: binucleates (anaphase) peak at a phase angle of 238°; septation peaks at a phase angle of 277°; and histone expression (S phase) peaks at a phase angle of about 312. Thus 0° is early in G2, but not the very beginning of G2. “ElutA_Fourier_component” is related to the amplitude of the gene's oscillation. It is the magnitude from the Fourier decomposition of the elutriation A data series. It is the magnitude of only one constituent waveform (the once-per-cell-cycle wave). It is in log_2_ space. “Combined_P” is the combined *p-*value obtained by using Stouffer's method to combine the *z* scores from elutriation A, elutriation B, and the cdc25 block release. “ElutA_Z,” ElutB_Z,” and “cdc25_Z” are the *z* scores for the elutriation A, elutriation B, and *cdc25* block-release experiments, respectively. *Z* scores were calculated from Monte Carlo simulations (see Materials and Methods). “All names” are all other known synonyms for the gene in question other than the “SUID” and the “Common-name.” In some cells of the spreadsheet, the entry is “#N/A” or “X” or “Z.” These entries indicate that a result was not calculated because of excessive missing data.
(2.9 MB XLS).

Table S2 SpikeChart Weight Matrices The weight matrices and spike height rules used by SpikeChart to generate Figure 10 are shown.
(24 KB DOC).

Table S3 *S. cerevisiae* Homologs of *S. pombe* Cell Cycle Genes For the top 200 *S. pombe* cell cycle genes, the best homologs in *S. cerevisiae* (if any) are shown. If the *S. cerevisiae* homolog oscillates through the cell cycle, then the time of peak expression is shown in the “Sc peak” column; if the homolog is not known to oscillate, then this column is marked “ND.” Any transcription factors thought to regulate the *S. cerevisiae* homolog are noted. If there are more than two *S. cerevisiae* homologs, then all these additional homologs are combined in a single field in the right-most column.
(53 KB XLS).

## Abbreviations

bp: base pair
