# Supplementary material for: The Cell Cycle–Regulated Genes of Schizosaccharomyces pombe
Source: PLoS Biol. 2005 Jun 28;3(7):e225. doi: 10.1371/journal.pbio.0030225 (PMC1157095; doi:10.1371/journal.pbio.0030225)
Supplement: Supplementary file 2 [file pbio.0030225.st002.doc]

```
Supplementary Table XX.  Weight Matricies for SpikeChart

Values at each position are multiplied to achieve the final score.

FKH
Consensus:  TGTAAACAAA

Weight A	1	0	0	4	4	4	0	4	4	4
Weight T	2	0	4	0	0	0	2	0	0	2
Weight C	1	0	0	0	0	0	4	0	0	1
Weight G	1	4	0	0	0	0	0	0	0	1

	Score			Height
	32,768			6
	65,536			7
	131,072			8
	262,144			9
	524,288			10


Ace2
Consensus:  ACCAGCCT

Weight A	4	0	0	4	0	0	1	2
Weight T	1	0	0	0	0	0	1	2
Weight C	1	4	4	0	0	4	8	1
Weight G	2	0	0	0	4	0	1	1

Score				Height
1024				5
2048				6
4096				7
8192				8
16,384			9
>16,384			10


Cdc10
Consensus:  GACGCGTC

Weight A	2	4	0	0	0	0	2	1
Weight T	1	0	0	0	0	0	2	1
Weight C	1	0	4	0	4	0	1	2
Weight G	4	0	0	4	0	4	1	1

	Score			Height
	1,024			6
	2,048			7
	4,096			8
	8,192			9
	>8,192		10


Dbl10
Consensus:  ACGCG(a/t)CGCG

Weight A	4	0	0	0	0	4	0	0	0	0
Weight T	1	0	0	0	0	4	0	0	0	0
Weight C	1	4	0	4	0.	1	4	0	4	0
Weight G	2	0	4	0	4	1	0	4	0	4


Score				Height
65,536			6
131,072			7
262,144			8
524,288			9
>524,288			10


New 1v.

Consensus:  (a/t)TG(A/t)CAAC.

	(a/t)	T	G	A/t	C	A	A	C

A	8	0	0	2	0	2	2	0

T	8	2	0	1	0	0	0	0

C	1	0	0	0	2	0	0	2

G	1	0	2	0	0	0	0	0


Score			Spike height
0				0
64				0
128				6
512				7
1024				10


New 3v.
New 3v consensus: (a/t) A C C (a/t) C G (t/c) (a/t) (c/a) C

A	2	2	0	0	2	0	0	0	1	2	1
T	2	1	0	0	4	0	0	1	2	1	1
C	1	1	1	1	0	1	0	1	0	4	2
G	1	1	0	0	0	0	1	0	0	1	1

Score		Spike Height
2		3
4		4
8		5
16		6
32		7
64		8
128		9
256		10
```
